# Characterization of the *Neisseria meningitidis* Helicase RecG

**DOI:** 10.1371/journal.pone.0164588

**Published:** 2016-10-13

**Authors:** Getachew Tesfaye Beyene, Seetha V. Balasingham, Stephan A. Frye, Amine Namouchi, Håvard Homberset, Shewit Kalayou, Tahira Riaz, Tone Tønjum

**Affiliations:** 1 Department of Microbiology, University of Oslo, Oslo, Norway; 2 Department of Microbiology, Oslo University Hospital (Rikshospitalet), Oslo, Norway; University of Massachusetts Medical School, UNITED STATES

## Abstract

*Neisseria meningitidis* (Nm) is a Gram-negative oral commensal that opportunistically can cause septicaemia and/or meningitis. Here, we overexpressed, purified and characterized the Nm DNA repair/recombination helicase RecG (RecG_Nm_) and examined its role during genotoxic stress. RecG_Nm_ possessed ATP-dependent DNA binding and unwinding activities *in vitro* on a variety of DNA model substrates including a Holliday junction (HJ). Database searching of the Nm genomes identified 49 single nucleotide polymorphisms (SNPs) in the *recG*_Nm_ including 37 non-synonymous SNPs (nsSNPs), and 7 of the nsSNPs were located in the codons for conserved active site residues of RecG_Nm_. A transient reduction in transformation of DNA was observed in the Nm *ΔrecG* strain as compared to the wildtype. The gene encoding *recG*_Nm_ also contained an unusually high number of the DNA uptake sequence (DUS) that facilitate transformation in neisserial species. The differentially abundant protein profiles of the Nm wildtype and *ΔrecG* strains suggest that expression of RecG_Nm_ might be linked to expression of other proteins involved in DNA repair, recombination and replication, pilus biogenesis, glycan biosynthesis and ribosomal activity. This might explain the growth defect that was observed in the Nm *ΔrecG* null mutant.

## Introduction

*Neisseria meningitidis* (Nm), or the meningococcus, is a Gram-negative bacterium that frequently colonizes the human oropharynx. In individuals who lack bactericidal antibodies, Nm can enter the bloodstream, cross the blood-brain barrier, and cause septicaemia and/or meningitis [1]. We are interested in how Nm cells survive on the oral mucosal surface, in the bloodstream and at the meninges, where it is exposed to reactive oxygen and nitrogen species that are typically highly genotoxic [2]. DNA repair pathways that promote genome stability and protect against oxidative DNA damage have been extensively characterized in *Escherichia coli*; however, the comparable DNA repair pathways in Nm are less well studied. It has been reported that *Neisseria* may be less proficient in DNA base excision repair (BER) than *E*. *coli* and also lacks an SOS response to DNA damage [2,3]. These features, and its genetic tractability due to its constitutive competence for transformation and short generation time, make Nm an excellent organism for investigating DNA repair mechanisms and pathways in a host-adapted pathogen [4].

Helicases play major roles in genome maintenance including repair, recombination and replication of DNA in all kingdoms of life. Due to the complexity of the DNA damage responses, very little is known about how helicase-dependent DNA repair pathways are regulated and coordinated with cell cycle checkpoints [5]. RecG is ubiquitous among bacterial species [6, 7], vascular plants and green algae [7] where it is targeted to mitochondria and chloroplast [8], however, homologues of RecG have not been detected in other eukaryotes or archaea. Bacterial RecG has two RecA-like helicase domains, an N-terminal wedge-containing domain and a C-terminal TRG (translocation by RecG) motif [9]. The RecG protein in *E*. *coli* (RecG_Ec_), which is extensively studied by Lloyd and co-workers, inhibits inappropriate DNA replication and aberrant chromosome segregation in cells exposed to UV irradiation [10]. RecG is also essential in *E*. *coli* cells lacking 3′ single-stranded DNA exonucleases to counteract PriA helicase-mediated DNA re-replication [11]. Homologous recombination is a fundamental cellular process that rearranges genes within and between chromosomes, promotes DNA repair and guides segregation of chromosomes [12]. In bacteria, RecG and RuvAB play critical roles in processing HJs and promoting branch migration [13]. RecG-deficient bacterial cells exhibit complex and variable phenotypes, including defects in transformation and pilin antigenic variation in Nm [14,15], defective growth and reduced radio-resistance in *Deinococcus radiodurans* [16], sensitivity to oxidative stress in *Pseudomonas aeruginosa* [17], and sensitivity to UV radiation in *P*. *aeruginosa* and Nm [14,17].

In this study, RecG_Nm_ was characterized and its roles in DNA recombination, repair, replication and transformation explored. Recombinant RecG_Nm_ was assessed for its DNA binding and unwinding activities on model DNA substrates in the presence and absence of ATP. Nm wildtype and Δ*recG*_Nm_ cells were compared with respect to cellular phenotype, response to genotoxic stress and protein expression signatures. The results provide insight into the possible biological roles of RecG_Nm_.

## Materials and Methods

### Cloning of the *N*. *meningitidis recG* and *ssb* genes

The Nm *recG* and *ssb* genes were PCR amplified from genomic DNA isolated from Nm strain MC58 using the primers listed in S1 Table. In brief, the ORF encoding *recG*_Nm_ in Nm strain MC58 was amplified by PCR using the primers GTB3 and GTB5 (S1 Table). The PCR product was cloned into the pET28b (+) plasmid (Novagen). The resulting plasmid, pGTB1, with an N-terminal 6xHis-tag, was transformed into *E*.*coli* ER2566. A construct pGTB1K294A bearing a point mutation in the ATP binding motif (K294A) was created from pGTB1 by site-directed mutagenesis using primers GTB17 and GTB18 (S1 Table). The Nm *ssb* gene cloning was performed as previously described [18]. For the construction of *ssb*NmΔC8 expressing a C-terminally truncated SSB_Nm_ protein, primers SF275 and SF276 were used to amplify the vector pSAF104 using the vector pEH1 as a template (S1 Table). The PCR product was joined by Gibson assembly [19] and, transformed into *E*. *coli*. Constructs were verified by sequencing.

### Overexpression, purification and characterization of recombinant proteins

The recombinant RecG_Nm_ was overexpressed in *E*. *coli* and the RecG_Nm_ protein was purified to homogeneity (S1 Fig). Briefly, the *E*. *coli* ER2566 cells harbouring plasmid pGTB1and pGTB1K294A were grown at 37°C in LB medium containing 50 μg/ml kanamycin until OD_600nm_ ≈ 0.4, the temperature was reduced to 18°C. Protein expression was induced with 0.5 mM isopropyl *β*-D-thiogalactopyranoside (IPTG) overnight. Cells were harvested by centrifugation and resuspended in lysis buffer, disrupted by sonication and the lysates were used as source material to purify RecG_Nm_ by affinity chromatography on Ni-NTA followed by gel filtration on Superdex 75. The SSB_Nm_ and SSB_NmΔC8_ proteins were purified as previously described [18].

### Model DNA substrate preparation and DNA binding, unwinding and ATPase assays

#### Preparation of DNA substrates

**Fig 1:**
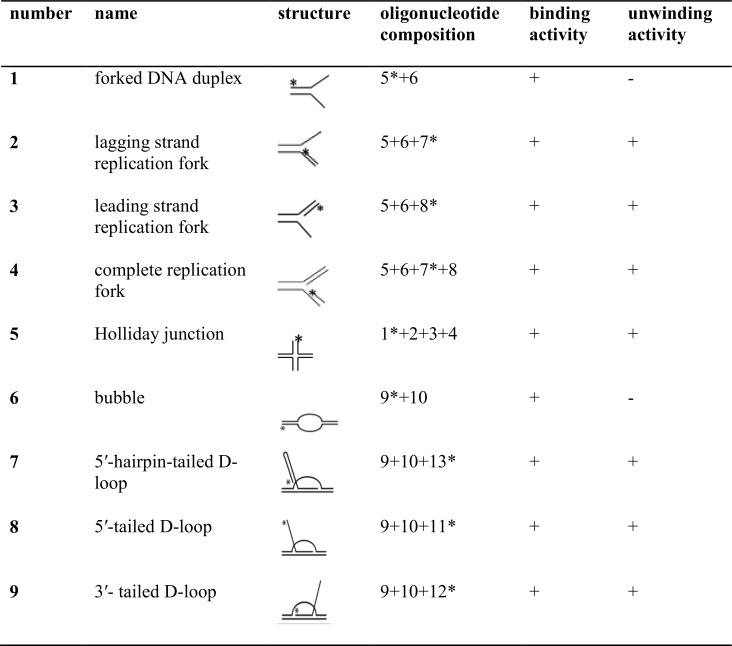
Schematic diagram of model DNA substrates, and the RecG_Nm_ DNA binding and unwinding activity. Minus and plus symbols indicate the absence or presence of RecG_Nm_ activity on the indicated substrate, respectively.

DNA oligonucleotides used in this study to generate model DNA substrates were adapted from previous studies [7,20–22]. DNA substrates were prepared essentially as described in [7]. Briefly, oligonucleotides were 5′-end labelled using γ-^32^P[ATP] (PerkinElmer) and T4 PNK enzyme (NEB) for 1 h at 37°C. Unincorporated ATPs were removed using illustra Microspin™ G-25 columns (GE Healthcare). Labelled and unlabelled complementary oligonucleotides were mixed at a molar ratio of 1:2.5, in annealing buffer [40 mM Tris-HCl (pH 8.0), 50 mM NaCl] and denatured at 95°C for 5 min and allowed to cool down to room temperature overnight. The annealed products were resolved on 8% non-denaturing polyacrylamide gel. The bands containing the completely annealed substrates were excised and DNA was eluted into [10 mM Tris-HCl (pH 8.0), 0.5 mM EDTA] buffer overnight at 4°C. The concentrations of the eluted DNA substrates were estimated as described elsewhere in [23]. For the ATPase assay, branched DNA substrates were prepared as indicated above except that the complementary oligonucleotides were not labelled with γ-32P[ATP] and the annealed products were not gel purified as previously described in [24]. The schematic diagram of the DNA substrates and DNA sequences are presented in Fig 1 and S2 Table, respectively.

#### Band shift assay

Band shift assay was carried out as described in [7]. Reaction mixtures (20μl) contained 0.1 nM γ-^32^P-labelled DNA substrates, binding buffer [40 mM Tris-HCl, (pH 8), 2.5 mM EDTA, 2 mM MgCl2, 100 mg/ml bovine serum albumin (BSA), 6% glycerol, and 1 mM DTT] and the indicated concentrations of the RecG_Nm_ or RecG_Nm_K294A protein. After incubation for 15 min on ice, 2 μl of 60% glycerol was added to the reaction immediately before loading on to a 30 min pre-run 5% native PAGE gel (29:1, acrylamide: bisacrylamide). Electrophoresis was done using low ionic strength buffer (6.7 mM Tris HCl pH 8, 3.3 mM sodium-acetate pH 5.5 and 2 mM EDTA pH 8) at 200V for 5 min followed by 160 V for 85 min in ice water bath with continuous buffer recirculation between the upper and lower chambers. Gels were dried using GD 2000 Vacuum Gel Dryer (Hoefer®, Inc). The products were visualized using Typhoon PhosphorImager, and the gel bands were quantitated using ImageQuant TL v2003.02 (GE Healthcare). Percent DNA bound was calculated as follows: percent DNA bound = (B/ (B+F)) x 100, where B is the bound DNA and F is the free DNA.

#### Helicase assays

All helicase unwinding reactions (10μl) were carried out in helicase reaction buffer [20 mM Tris-HCl (pH 7.5), 50 mM NaCl, 1 mM DTT, 2 mM MgCl_2_, 2 mM ATP and 50 mg/ml BSA]. 0.1 nM γ-^32^P-labeled DNA substrate was mixed with increasing concentration of RecG_Nm_ or RecG_Nm_K294A and incubated at 37°C for 30 min. The reaction was terminated by adding 5μl of 3x stop dye (50 mM EDTA, 40% glycerol, 0.9% SDS, 0.1% bromophenol blue and 0.1% xylene cyanol) along with 10x molar excess unlabeled oligonucleotide complementary to the unlabeled strand in the substrate. The reaction products were analyzed on 8% native polyacrylamide (19:1) gel containing 0.1% SDS in 1x Tris/borate/EDTA buffer. Gels were dried, exposed, visualized and quantitated as described for DNA binding assay. Percent helicase unwound was calculated as follows: percent unwound = (P/(S+P)) x 100, where P is the product and S is the residual substrate. Values of P and S were determined by subtracting background values in controls having no enzyme and heat denatured substrate, respectively. The Kd value of the data obtained from binding and unwinding assay was analysed using GraphPad Prism 5 with curve fitting using nonlinear regression.

#### ATPase assay

RecG_Nm_ ATP hydrolysis activity was monitored by thin-layer chromatography (TLC), as previously described [7]. RecG_Nm_ or RecG_Nm_K294A was added to initiate a 10 μl reaction in the presence of 100nM DNA cofactor in ATPase buffer [20 mM Tris/HCl (pH 7.5), 2 mM MgCl_2_, 100 μg BSA/ml, 25 mM cold ATP, 0.023 nM [γ-^32^P]ATP, 2 mM DTT]. Also reactions containing DNA cofactor but without the wild type (RecG_Nm_) protein, and RecG_Nm_ but without DNA cofactor were included per experiment. The reaction mixture was incubated at 37°C for the indicated times and terminated by adding 5 μl of 0.5 M EDTA (pH 8.0). Samples (2 μl) were spotted onto TLC plates (PEI Cellulose F, Merck) at 1.5 cm intervals and resolved using a solution containing 1 M formic acid and 0.5 M LiCl. The TLC plates were air-dried, exposed to a phosphorimaging screen, imaged and quantified as described above for the DNA binding assays. The percentage of hydrolyzed ATP was calculated as {counts for γ-32Pi / (counts for γ-32Pi+counts for [γ-32P]ATP)} x 100. The values obtained from samples lacking RecG_Nm_ were subtracted from the samples containing RecG_Nm_ to account for background ATP hydrolysis.

### Construction of an *N*. *meningitidis ΔrecG* mutant

The *recG* DNA fragments were designed to recombine and integrate into the host chromosome allowing the *recG* gene to be interrupted by an antibiotic resistance gene. For this purpose, primer pairs SF81/SF82 and SF83/SF84 were used to amplify two regions covering bp 343–821 and bp 893–1439 of the *recG* gene, respectively. These were then ligated with a kanamycin resistance gene (*aph*) and the pBluescriptIISK+ vector (Stratagene) by 4-point ligation. The resulting plasmid, pSAF48, conferring resistance to ampicillin and kanamycin, was transformed into XL1-Blue (Stratagene) for plasmid propagation. The sequence of the insertion was verified by DNA sequencing. The plasmid was transformed into Nm strains MC58, M1080 and M400 and *Neisseria gonorrhoeae* (Ng) strains MS11 by natural transformation using kanamycin resistance as selective marker for null mutants.

### Bacterial strains and growth conditions

The bacterial strains and plasmids employed in this study are listed in Table 1. Neisserial strains were grown on GC agar plates or in liquid GC medium (7.5 g/l peptone, 3.75 g/l tryptone, 4 g/l K_2_HPO_4_, 1 g/l KH_2_PO_4_, 5 g/l NaCl) supplemented with IsoVitaleX at 37°C and 5% CO_2_. When required, kanamycin at a final concentration of 100 mg/l was added. *E*. *coli* was grown in LB medium or on LB plates containing kanamycin (50 mg/l) at 37°C. Nm wildtype and Nm Δ*recG* mutant strains were grown at 34°C in 5% CO_2_ for 18–24 hours. Growth properties were assessed by comparing colony edges (sharp or diffuse) [25], colony size and colony number on GC plates with 1% agar. Pictures of meningococcal colonies were taken using a stereo microscope (Leica) equipped with a CCD camera.

**Table 1:** Bacterial strains and plasmid constructs employed in this study.

	Relevant characteristic	Source
**Plasmids**		
**pET28b(+)**	bacterial expression vector with T7 promoter; kanamycin resistance	Novagen
**pGTB1**	pET28b(+) based vector with *recG*_*Nm*_ insert between *Xho*I and *Nde*I	This study
**pGTB1K294A**	pGTB1 based vector with site directed mutant of *recG*_Nm_ in motif I	This study
**pDV-c-d1**	pBluescriptIISK+ based vector with *pilG*::*Erm*^*r*^ and DUS	[27]
**pEH1**	pQE-30 harboring *ssb* from Nm MC58	[18]
**pSAF104**	pEH1-based plasmid harboring *ssb*NmΔC8	This study
**Strains**		
***Escherichia coli***		
**ER2566**	*fhuA2 lacZ*::T7 gene1 [lon] ompT gal *sulA11* R(mcr-73::miniTn10—Tet^S^) 2 [dcm] R(zgb-210::Tn10—Tet^S^) *endA1* Δ(*mcrC*-mrr)114::IS10.	New England Biolabs
**XL-1 Blue**	*recA1 endA1 gyrA96 thi-1 hsdR17 supE44 relA1 lac* [F´ *proAB lacI*q*Z*Δ*M15* Tn*10* (TetR)]	Stratagene
***Neisseria meningitidis***		
**MC58**	serogroup B, isolated in England	[28]
**MC58Δ*recG***	derivative of MC58 with *recG*::*Kan*^*r*^	This study
**M1080**	serogroup B, isolated in the United States in 1984	[29]
**M1080Δ*recG***	derivative of M1080 with *recG*::*aph*; kanamycin resistance	This study
**M400**	derivative of M1080 containing the IPTG -inducible *recA6* allele (TetM)	[30]
***Neisseria gonorrhoeae***		
**MS11**		Herman Schneider
**MS11Δ*recG***	Derivative of MS11 with *recG*::*aph;* kanamycin resistance	This study

For colony size measurement, overnight grown Nm wildtype and Nm *ΔrecG* mutant cells were suspended in liquid GC medium and adjusted to OD_660_ = 0.2. A tenfold serial dilutions of the cells were prepared in 1x PBS and 50 μl aliquots of the 10^−6^ dilutions were spread on GC agar plates. The plates were incubated with 5% CO_2_ at 37°C for 18 hours. Pictures of whole plates were captured using a Lifecam camera (Microsoft) at a resolution of 8 megapixels. Colony count and colony size measurements were performed using the OpenCFU 3.8 BETA software [26] with settings for the minimum radius set to 2 pixels and the maximum radius set to Auto-Max.

### Quantitative transformation assay

Quantitative transformation was performed as previously described [14,31] using plasmid pDV-c-d1 carrying an antibiotic resistance marker. Briefly, Nm cells were pre-grown on GC plates overnight at 37°C and resuspended in 5% CO_2_ saturated GC medium containing IsoVitaleX and 7 mM MgCl_2_. 5 μl of DNA (100 ng/μl) were provided in 15 ml tubes, 500 μl cell suspension was added, mixed and incubated at 37°C for 15 min without agitation followed by the addition of 25 U/ml benzonase and incubated at 37°C for 10 min to degrade extracellular DNA. Samples were diluted by adding 4.5 ml GC medium and incubated for 4.5 h at 37°C on a rotator drum at 60 rpm. Of each undiluted sample, 50 μl aliquots were spread on GC agar plates containing 8 mg/l erythromycin and 100 μl of 10^−5^ and 10^−6^ dilutions, prepared in PBS, were spread on GC agar plates without antibiotics. Following overnight incubation at 5% CO_2_ and 37°C colonies were counted. Transformation frequency was calculated as the number of antibiotic-resistant colony forming units (CFU) per total CFU.

### Spontaneous mutation assay

Spontaneous mutation rates were determined as previously described [4,32] with minor modifications. Briefly, overnight grown Nm wildtype and *ΔrecG*_*Nm*_ cells were suspended in GC medium with the OD_660_ adjusted to 0.02. The suspension was further diluted 10 fold and the cells were grown at 37°C for 6 hours. Then, 50 μl of the undiluted and 10^−1^ diluted cells were spread on GC plates containing 3 mg/l rifampicin, whereas 10^−5^, 10^−6^, and 10^−7^ dilutions were spread on plain GC plates. The cells were grown for 24 hours at 37°C and 5% CO_2_ and the colonies were counted. The mutation rate was calculated as a ratio of rifampicin-resistant colony forming unit (CFU) to the total number of CFU. The assay was repeated 5 times for each strain.

### SDS-PAGE and immunoblotting

Procedures for sample preparation, SDS-PAGE and antigen detection have been described previously [33,34]. The presence of RecG, SSB and pilin, respectively, in Nm whole-cell lysates was detected by immunoblotting using rabbit polyclonal antiserum raised against recombinant RecG_Nm_ and SSB_Nm_ and purified Nm pili.

### Flow cytometry analysis

Nm can cause serious systemic infections [35], therefore, the relatively less invasive pathogen Ng was used to perform flow cytometry analysis outside the neisseria (biosafety level-2) laboratory. Colonies of Ng MS11 wildtype and Ng *ΔrecG* grown for 20–24 hours were suspended in CO_2_-saturated liquid GC medium supplemented with IsoVitaleX to OD_660_ ≈ 0.02. The cell suspension was diluted 10 times with GC medium and cells grown at 37°C overnight at 30 rpm to OD_660_ ≈ 0.16. The cultures were further diluted 10 times and cells grown at 37°C for 4 doubling times at 60 rpm until OD_660_ = 0.14–0.18. Ng has a doubling time of 60 min at 37°C [36]. A 1 ml sample from the exponentially growing cultures of non-treated cells was collected and kept on ice until further processing. To 3 ml exponentially growing Ng cells, rifampicin (36 μg/ml) [37] and cephalexin (4 μg/ml) [38] were added, and cells were allowed to grow for additional six doubling times. Rifampicin inhibits initiation of replication but allows the current round of replication to continue to completion (replication runout), resulting in fully replicated chromosomes. Cephalexin stops cell division, resulting in integer numbers of chromosomes per cell [36,39]. Both, treated cells and non-treated control cells, were further processed as described elsewhere [40,41]. Briefly, the cells were pelleted at 14000×g for 4 min at 4°C, washed in TE buffer, resuspended in 100 μl TE buffer and fixed by addition of 900 μl 77% ethanol and incubated overnight. The fixed cells were washed in 1 ml ice cold 0.1M phosphate buffer (PB) and resuspended in 500 μl PB. The cells were stained with 1.5 μg/ml fluorescein isothiocyanate (FITC) in PB at 4°C overnight, washed in 1 ml ice-cold 0.02 M Tris-buffered saline (TBS; 20 mM Tris-HCl pH 7.5, 130 mM NaCl, pH 7.5). The cells were resuspended in 500 μl TBS with 1.5 μg/ ml Hoechst 33258 and kept for 30 minutes_._ Stained cells were passed through a 5 μm syringe Filter (Pall Life Sciences). To investigate the DNA content and chromosomal DNA replication patterns, slowly growing *E*. *coli* CM735, the majority containing one or two copies of chromosomal DNA [40], were used as standard to calibrate the flow cytometer. Sample processing was carried out as previously described [40] on a BD LSR II flow cytometer (BD Biosciences), and the data obtained from the flow cytometer were analysed using FlowJo version 10 software.

### Genotoxic stress assays

Nm cells from overnight plate culture were suspended in liquid GC medium to OD_660_ = 0.3 and diluted 10 fold in CO_2_ saturated GC medium containing IsoVitalex. The cells were allowed to grow for two hours at 37°C with rotation. 990 μl of the cells suspension was mixed with 10μl of 10 mM hydrogen peroxide, 50 mM paraquat, 1M MMS or 1μg/ml MMC. After the cells were grown for one additional hour with rotation at 37°C, 50 μl aliquots of 10^−5^ and 10^−6^ dilutions in PBS were spread on GC agar plates. To test sensitivity to ultraviolet radiation, 50 μl aliquots of 10^−5^ and 10^−6^ dilutions of non-treated cells were spread on GC agar plates, irradiated at UV intensities of 0–80 J/m^2^ by using a CL-1000 Ultraviolet cross linker (Upland America). Finally, the plates were incubated overnight at 37°C with 5% CO_2_ for 12 to 18 hours. Colonies were counted and survival rate was calculated as the ratio of the number of colony forming units (CFU) from treated to non-treated samples.

### Bioinformatics analysis

Sequence data for alignment of the *recG* gene from *Neisseria* members was obtained from NCBI [42]. The Nm *recG* nucleotide sequences were searched for occurrences of the DNA uptake sequence (DUS) and single nucleotide polymorphisms, and the deduced RecG_Nm_ amino acid sequence was searched for predicted structural motifs. The orientation of DUS was determined using The Sequence Manipulation Suite [43]. SNP analysis of the *recG* gene among 14 Nm strains available at Genbank was conducted using MEGA6 [44]. In the SNP analysis, only the first and the second codon positions were considered.

For the RecG 3D homology model, the sequence conservation was calculated from all available variants of NEIS0433 using plotcon from the EMBOSS package [45]. The variability was visualized colour coded on the protein structure using ConSurf [46]. The Phyre2 service [47] was used to predict the 3D structure of the protein.

### Proteomic analyses

i) Sample pre-treatment: Nm wildtype and *ΔrecG* cells of strain MC58 were harvested from GC agar plates. The cells were washed three times in PBS, inactivated at 60°C for 30 min, and resuspended in 2% SDS/10mM Tris-HCl, pH7.5 containing EDTA free protease inhibitor cocktail (Roche) and PhosStop (Roche). The samples were transferred to Lysing Matrix B tubes (Roche) and disrupted in a MagNa Lyser (Roche). The supernatant was collected and the protein concentration measured by Direct Detect (Millipore). Per sample 100μg of protein lysate was separated on 4–12% Bis-Tris polyacrylamide gel (Life technologies). Each gel lane was separated and divided into 6 pieces and the samples reduced with DTT (Sigma-Aldrich) followed by alkylation with iodoacetamide (Sigma-Aldrich) and in-gel digest with trypsin (Promega). The peptides were extracted from the gel pieces with acetonitrile and purified on C_18_ ZipTip prior to nLC-MS/MS analysis. ii) Mass spectrometry. Samples were injected into an EASY 1000 nLC (Thermo Scientific) coupled to a Q-Exactive MS (Thermo Scientific) using a data-dependent Top10 method. A two-column set up was used with pre-column (Acclaim PepMap 100, 75μm × 2cm, nanoviper, C18, 3μm, 100Å, Thermo Scientific) and analytical column (PepMap RSLC, C18, 2μm, 100Å, 50μm × 15cm, Thermo Scientific). Each sample was injected in triplicates. Peptides were separated using a 120 minutes gradient with solvent A (0.1% FA/3%ACN (FA:LC-MS grade, Fluka; ACN: LC-MS grade, Merck) and solvent B (0.1%FA/97% ACN) using the following steps: I) 2% to 30% B from start to 90 min, II) 30% to 45% B from 90 min to 100 min, III) 45% to 90% B from 100 min to 115 min, IV) 90% B from 100 min to 120 min. iii) Database search and statistics: MS results were analysed using MaxQuant software version 2 against the proteome from Nm MC58 (UP000000425, Uniprot). T-test calculations were performed in Perseus version 1.2.0.17 using the label free quantitative (LFQ) values. Differentially expression with a p-value <0.05 was considered to be statistically significant.

All functional categories were obtained using the Kyoto Encyclopedia of Genes and Genomes (KEGG) using blastKOALA [48]. Briefly, an in-house python script was used to retrieve and blast the sequences of the identified proteins using blastKOALA. Proteins with existing KEGG pathway, module, or functional hierarchy (BRITE) annotations were identified. In addition, we used the Cluster of Orthologous Classification (COG) from the NCBI database for functional protein group annotations.

### Co-gel filtration interaction assay

The interaction between RecG_Nm,_ and SSB_Nm,_ and between RecG_Nm_ and SSB_NmΔC8_ was studied by gel filtration on a Superdex 200 10/300 GL column (GE Healthcare). Purified RecG_Nm_ protein was mixed independently with SSB_Nm_ and with SSB_NmΔC8_ proteins in a buffer consisting of 20 mM Tris pH (7.5), 600mM NaCl, and 1 mM DTT to a final volume of 300 μl. The samples were injected into a column equilibrated with the same buffer. The proteins were eluted in aliquots of 0.5 ml using the same buffer at 0.5 ml/min, and 13μl of each fraction was separated on SDS-PAGE and stained with Coomassie blue. The concentration of proteins used in the co-filtration assay was determined by DirectDetect (Millipore).

### Microscale thermophoresis

Microscale thermophoresis (MST), a method for measuring molecule interaction, is described extensively elsewhere [49]. Labelling of SSB_Nm_ was carried out following the manufacturers’ instructions using the Monolith NT Protein Labeling Kit RED–NHS (NanoTemper Technologies GmbH) resulting in a degree of labelling (DOL) of 0.65. Different concentrations of RecG_Nm_ were incubated with 21 nM SSB_Nm_ in 20 mM HEPES buffer (pH 7.5) containing 300 mM NaCl, 0.05% Tween 20, 0.1% Pluronic F-127, 0.1% PEG 8000 and 2 mM DTT. Samples were immediately loaded into Premium Coated capillaries (NanoTemper Technologies GmbH) and measured at 22°C and 40% MST power.

## Results

### RecG_Nm_ binds and unwinds DNA

**Fig 2:**
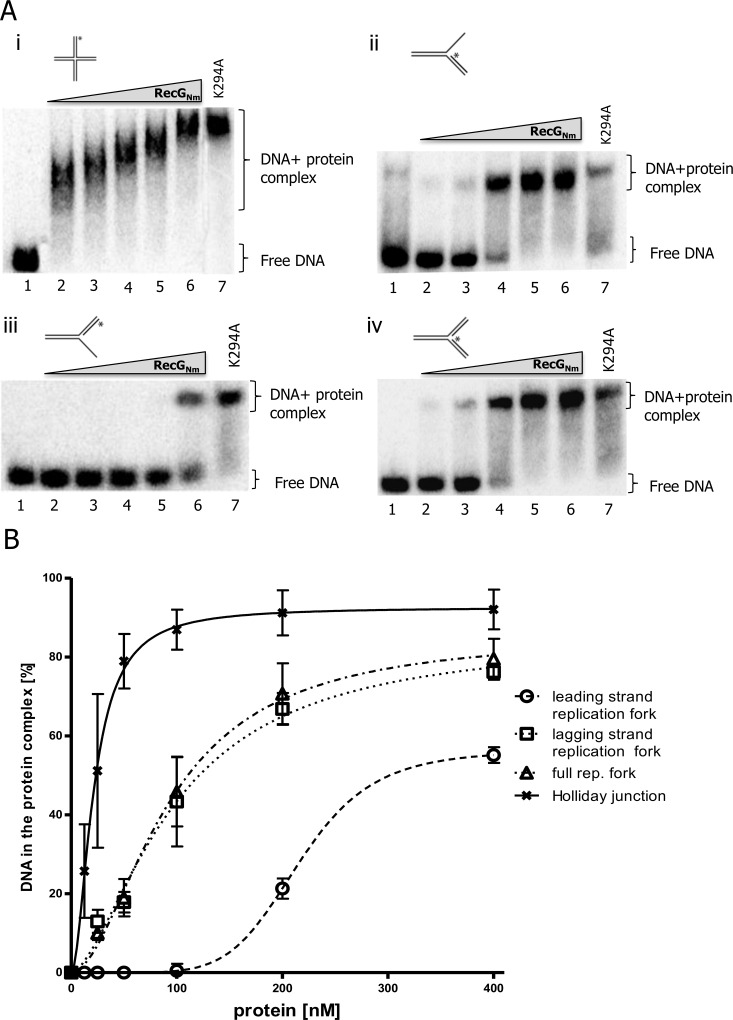
RecG_Nm_ binds different model DNA substrates. A. Representative gel images of DNA binding assays where increasing amounts of RecG_Nm_ was incubated with **i**. Holliday junction, ii. lagging strand replication fork, iii. leading strand replication fork, and iv). full replication fork. Lanes 1) reaction without protein; 2–6) 25, 50, 100, 200 and 400nM RecG_Nm_, respectively, 7) 400nM RecG_Nm_K294A. B. Quantitation of the gel images i, ii, iii, and iv in (A.) and the calculated Kd value for each Kd = 21.42, Kd = 97.79, Kd = 216.6, and Kd = 92.18, respectively. Data presented is mean ± SD from 3 independent experiments.

The DNA binding ability of RecG_Nm_ was investigated and using a band shift assay and DNA oligonucleotide substrates that resemble intermediates of DNA replication, repair and recombination. The experiments were performed with recombinant RecG_Nm_ and ATPase-deficient RecG_Nm_K294A. Both proteins showed equal ability to bind branched DNA substrates (Fig 2A and S1 Fig) and the HJ was the preferred substrate for binding (Fig 2B and S2 Fig). RecG_Nm_ and RecG_Nm_K294A also bind D-loop substrates containing a 5′-tail, 3′- tail or a hairpin-terminated tail (S3 Fig).

**Fig 3:**
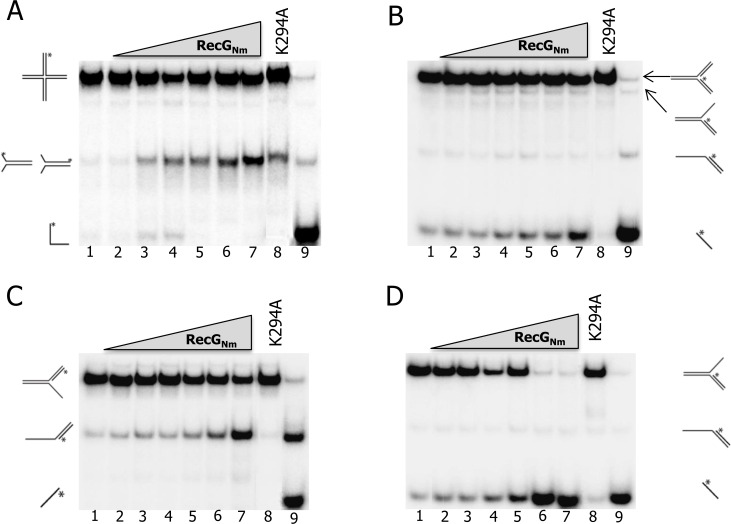
RecG_Nm_ branch-migrates Holliday junction and catalyses unwinding of replication forks. Gel images of DNA unwinding assays where increasing amounts of RecG_Nm_ was incubated with 0.1 nM A. Holliday junction, B. full replication fork, C. leading strand replication fork, D. lagging strand replication fork. Lanes: 1) reaction without enzyme, for Holliday junction substrate, 2–7) 12.5, 25, 50, 100, 200, and 400 nM RecG_Nm_, respectively, 8) 400 nM RecG_Nm_K294A; for fork substrates, 2–7) 1, 2, 4, 6, 12, and 25 nM RecG_Nm,_ respectively, 8) 25 nM RecG_Nm_K294A, 9) Boiled substrate.

To test the specific unwinding activity on branched DNA substrates_,_ increasing concentrations of RecG_Nm_ were incubated with end-labelled DNA substrates in the presence of 2 mM ATP and Mg^2+^. RecG_Nm_ promoted branch migration of a HJ substrate generating flayed duplexes (Fig 3A) and unwound both strands of a complete replication fork (Fig 3B). The unwinding activity of RecG_Nm_ was weaker on a leading strand replication fork than on a lagging strand replication fork (Fig 3C and 3D). RecG_Nm_ also unwound a D-loop with a 5′- tail, 3′-tail or a hairpin-tail (S4 Fig). RecG_Nm_K294A had no significant unwinding activity on any DNA substrate examined here (Fig 3).

### RecG_Nm_ is a DNA-dependent ATPase

**Fig 4:**
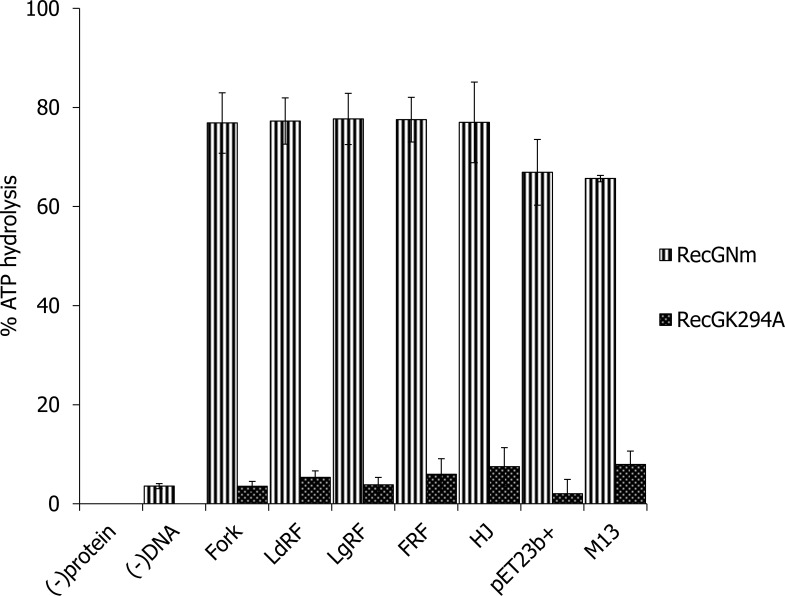
RecG_Nm_ is a DNA dependent ATPase. A graph showing ATPase activity of RecG from *Neisseria meningitidis* (RecG_Nm_) and RecG_Nm_K294A in the presence of DNA cofactors; forked DNA duplex, leading strand replication fork, lagging strand replication fork, HJ, M13mp18 ssDNA, and pET28b(+) dsDNA. % ATP hydrolysis is a measure of the percentage of inorganic phosphate released by the cleavage of the γ-phosphate of ATP. The standard deviations indicated by bars are from 3 independent experiments.

The ATP hydrolysing activity of recombinant RecG_Nm_ and RecG_Nm_K294A was investigated using branched DNA cofactors such as forked DNA duplex, leading strand replication fork, lagging strand replication fork, and HJ; including circular ssDNA, circular dsDNA, and homopolymeric oligonucleotides. RecG_Nm_ displayed comparable capacity of ATP hydrolysis in the presence of different types of branched DNA cofactors (Fig 1), as measured by the percentage of inorganic phosphate (≈ 77%) released by the cleavage of the γ-phosphate. The efficiency with which RecG_Nm_ hydrolysed ATP (≈ 67% of input ATP) in the presence of circular ss- or ds- DNA was marginally less than its ATPase activity with branched DNA cofactors, yet not statistically significant (Fig 4); whereas RecG_Nm_K294A nearly lost ATPase activity that is, < 8% of the input ATP hydrolysed in the presence of branched DNA, circular ssDNA and circular dsDNA cofactors (Fig 4 and S5A Fig). RecG_Nm_ also hydrolyzed ATP efficiently in the presence of homopolymeric ssDNA and dsDNA (S5B and S5C Fig), but RecG_Nm_K294A had very weak ATP hydrolysis activity in the presence of these DNA cofactors. The ATPase activity of RecG_Nm_ without DNA cofactor was almost non-detectable confirming that RecG_Nm_ is a DNA-dependent ATPase (Fig 4 and S5 Fig).

### Effect of Δ*recG*_*Nm*_ on colony morphology and colony size

**Fig 5:**
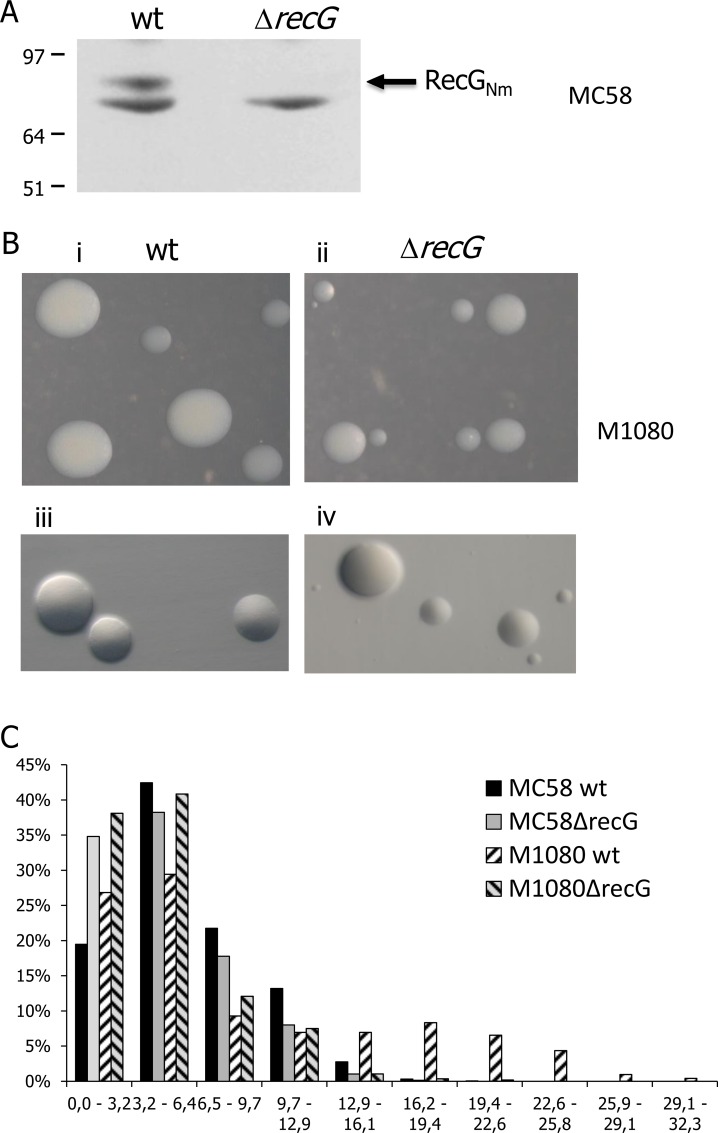
Phenotypic characterization of the Nm*ΔrecG* mutant. A. Immunoblot of cell lysates from *Neisseria meningitidis* (Nm) Mc58 wildtype and a Mc58Δ*recG* mutant detected with anti-RecG antiserum. B. Colony morphology of M1080 wildtype and M1080Δ*recG* null mutant of grown overnight at 34°C, showing normal size colonies for the wildtype (i and iii), whereas the mutant strain shows normal size colonies together with small size colonies (ii and iv). C. Graphical presentation of the colony size measurement (area in mm^2^) of the Nm wildtype strains and NmΔ*recG* Mc58 and M1080 mutant strains.

Immunoblot analysis with an antibody against RecG confirmed that the resulting Nm Δ*recG* strains lack the RecG protein (Fig 5A). The absence of RecG in the Nm Δ*recG* mutant was also confirmed by mass spectrometry. Growth on solid media was compared for the parental Nm strains MC58 and M1080 and the respective *ΔrecG* derivative strains. Nm MC58Δ*recG* and M1080Δ*recG* produced more small-sized colonies than the corresponding wildtype strains (Fig 5B). Large wildtype colonies were auto-agglutinating, while the small Δ*recG* colonies were not auto-agglutinating (Fig 5B). The average colony size was 6.8 (SD = 4.8) vs. 7.1 (SD = 6.9) mm^2^ for MC58 mutant vs. wildtype and for M1080 8.6 (SD = 7.4) mm^2^ and 4.5 (SD = 3.1) mm^2^ for MC1080 mutant vs. wildtype (Fig 5C). The proportion of colonies 0 to 3.2 mm^2^ was 19% for Nm MC58 wildtype and 3% for MC58 Δ*recG* and 27% and 38% for the M1080 wildtype and M1080 Δ*recG*, respectively.

**Fig 6:**
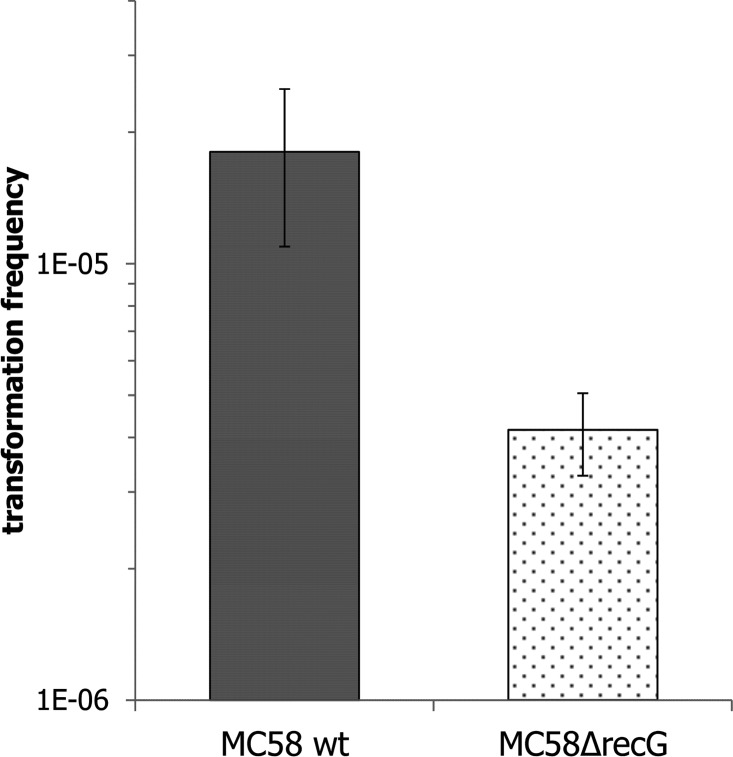
DNA transformation is reduced in *ΔrecG* mutant. Quantitative transformation of *Neisseria meningitides* with DUS-containing plasmid DNA. The standard deviations of the median from 3 independent experiments are indicated by bars. The values on the Y- axis are in logarithmic scale. Five agar plates were inoculated from each sample.

As previously reported for Ng [14], Nm strain MC58 Δ*recG* demonstrated reduced competence for transformation. The reduced competence was only transiently observed, just after DNA was added (Fig 6 and S6 Fig). No significant change in spontaneous mutation rate was observed in Nm Δ*recG* mutant strains. Spontaneous mutation rates of 2.4×10^−8^ (SD = 4.7×10^−8^) and 2.3×10^−8^ (SD = 4.7×10^−8^) were detected in Nm wildtype and Δ*recG*, respectively.

### DNA replication and the number of replication forks

**Fig 7:**
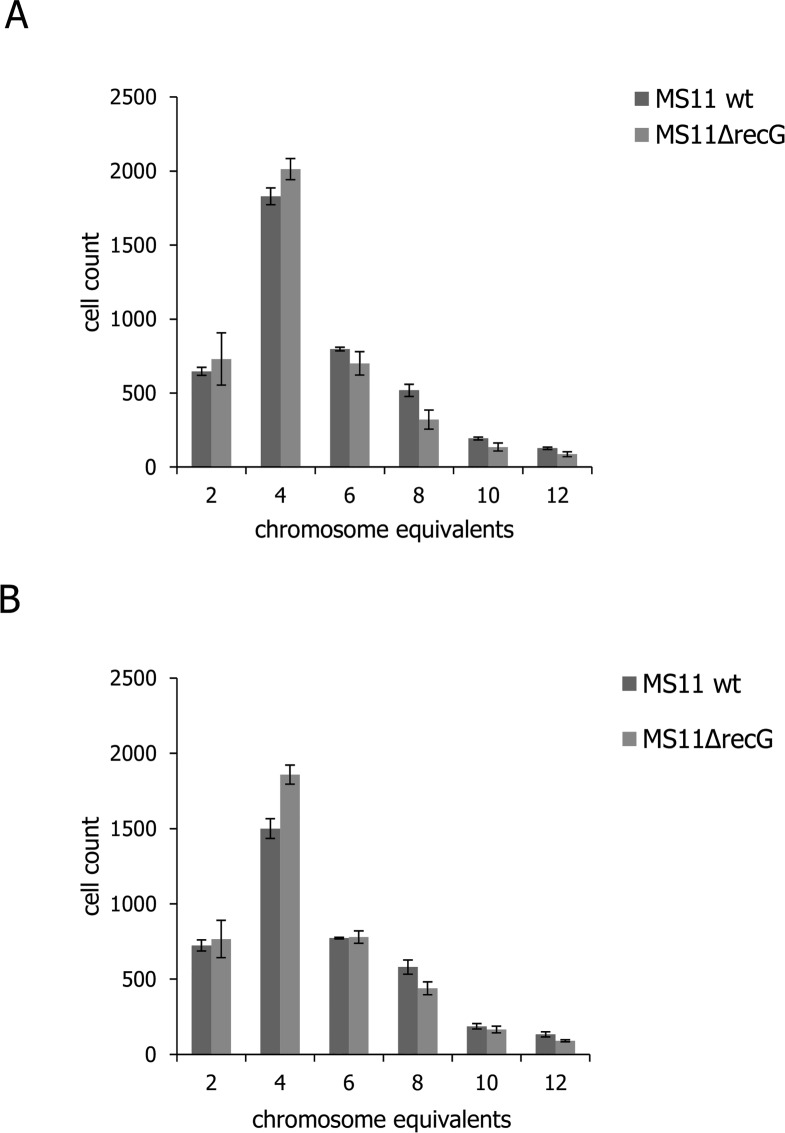
Distribution of *Neisseria gonorrhoeae* MS11 wildtype and ΔrecG mutant cells in flow cytometry analysis. Flow cytometry of Hoechst-stained, fixed bacterial cells was performed. The x-axis shows fluorescence levels, which indicates the amount of DNA content per particle counted. Genome equivalents were determined from the stationary phase and rif-treated cells. The graphs represent the distribution of MS11 wildtype and ΔrecG mutant strains, A. from the exponential culture, B. treated with rifampicin and cephalexin acquired by selecting/gating the sub-population of cells/particles with fluorescence level corresponding to chromosome equivalents of 2, 4, 6, 8, 10 and 12.

To estimate rate of cell growth and rate of DNA replication fork progression, total DNA content and total protein mass of Ng MS11 wildtype and Δ*recG* cells were measured. DNA content was measured from fluorescence intensity after staining with Hoechst 33258 and protein mass was estimated by performing flow cytometry on FITC-stained cells. The Ng MS11 wildtype and Δ*recG*_*Ng*_ mutant cells were used to estimate chromosome equivalents and the number of active replication forks per cell. For exponentially growing MS11 wildtype and Δ*recG*_*Ng*_, DNA content was 276 and 251 (fluorescent in arbitrary units, au), respectively. After treatment with rifampicin and CPX, DNA content was 277 and 262 au, respectively (S3 Table). Equal number of chromosome equivalents exists in MS11 wildtype and Δ*recG* mutant cells treated with rifampicin and CPX (Fig 7 and S7 Fig). This suggests no difference in the number of active replication forks in wildtype and mutant strains.

On the other hand, a slight difference was seen regarding the distribution of chromosome equivalents. For MS11Δ*recG*, 69% of the untreated and 64% of the treated cells contained 2 or 4 chromosome equivalents, while only 60% and 57%, respectively, of the wildtype cells contained 2 or 4 chromosome equivalents (Fig 7).

### Nm MC58 Δ*recG* mutant cells are sensitive to genotoxic agents

**Fig 8:**
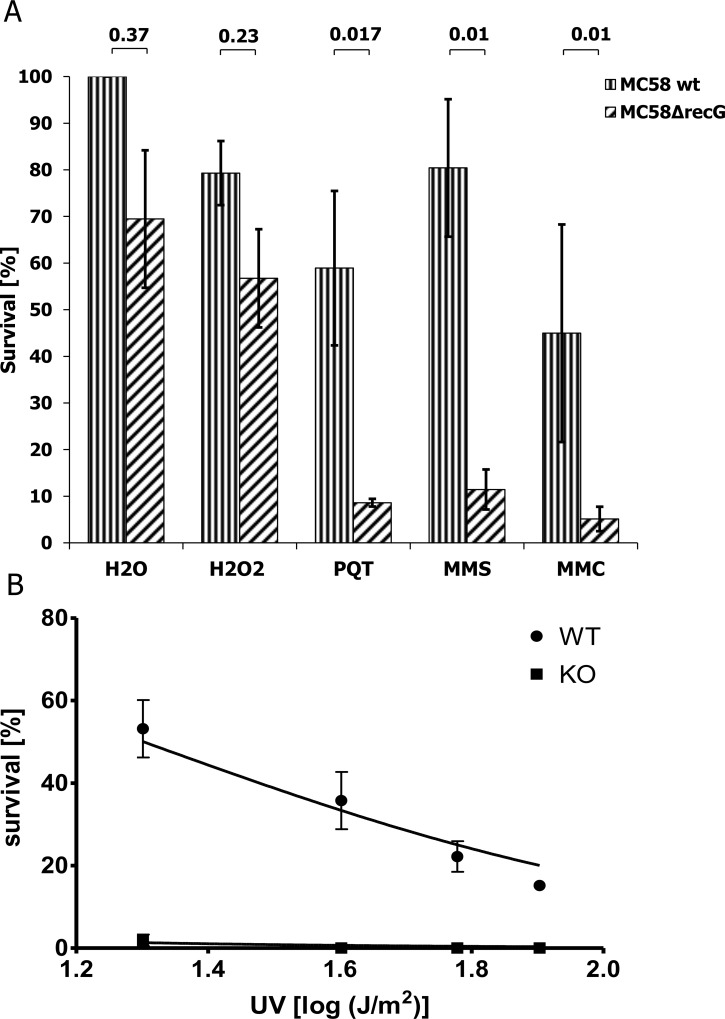
Alkylating agent, DNA cross linker and UV light affect the survival of a *Neisseria meningitidis* Δ*recG* mutant. A. *N*. *meningitidis* (Nm) MC58 wildtype and MC58Δ*recG* mutant were treated with hydrogen peroxide, paraquat, MMS and MMC. B. Survival rate of Nm MC58 wildtype and MC58Δ*recG* was determined after exposing the cells to the indicated UV influences. The standard deviations of the median from 3 independent experiments are indicated by bars.

Survival of Δ*recG*_*Nm*_ mutant was investigated in the presence of hydrogen peroxide, paraquat, MMS, MMC or UV radiation. Nm Δ*recG* cells were 6-fold more sensitive to paraquat, 7- and 8-fold more sensitive to MMS or MMC treatment than wildtype (Fig 8A) and also more sensitive to UV-irradiation than wildtype (2% survival vs 46% survival after exposure to 20 J/m^2^ UV. Higher doses of UV killed all Δ*recG* mutant cells (Fig 8B). Δ*recG*_*Nm*_ wildtype and mutant cells were approximately equally sensitive to hydrogen peroxide (Fig 8A).

### DNA uptake sequence and single nucleotide polymorphism in *recG* in the pathogenic Neisseria

**Fig 9:**
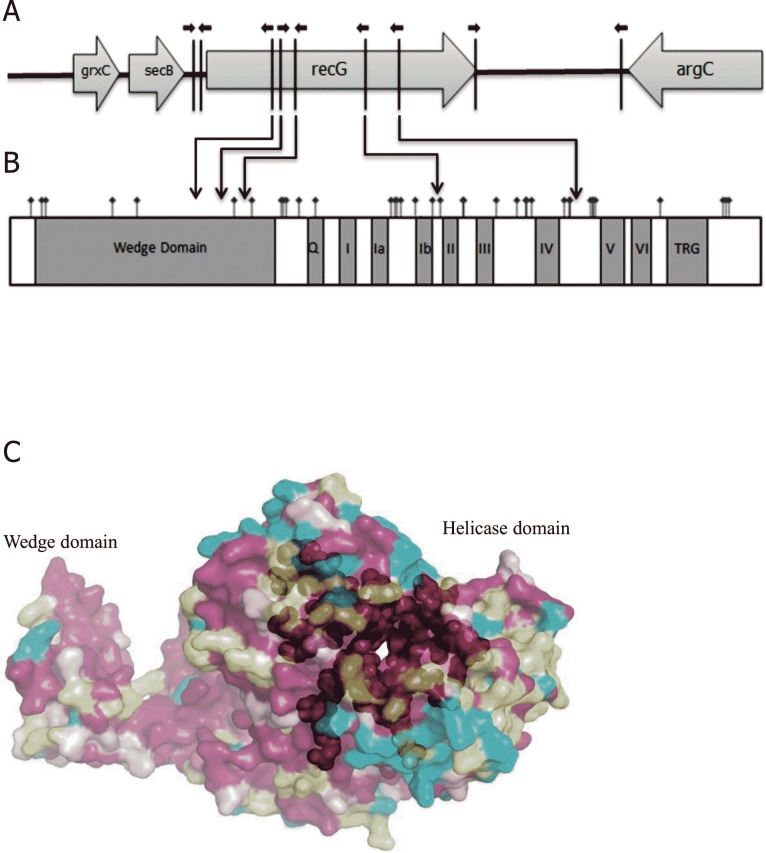
*Neisseria meningitidis recG* (*recG*_*Nm*_) is a DNA uptake sequence (DUS) abundant gene. A. Schematic diagram of the *N*. *meningitidis* (Nm) *recG* gene **(***recG*_*Nm*_) and neighbouring genes showing the position of the DNA uptake sequences (DUS) (black arrows). B. Domain organization in the RecG_Nm_ and non-synonymous single nucleotide polymorphism (nsSNP) identified in *recG*_*Nm*_ from different Nm strains. The positioning of the nsSNPs is shown in square tick marks (bold). C. The predicted structure of RecG_Nm_ with colour coding for conserved (red) and variable (blue) regions, yellow regions indicate insufficient data. The RecG_Nm_ regions outside of the helicase motifs are shown in transparent.

Neisserial genomes carry about 2000 copies of the 10 bp DUS motif, 5'-GCCGTCTGAA-3' [50–52], which facilitates DNA binding and uptake during genetic transformation between neisserial cells. At least one DUS in the donor DNA is required for efficient transformation of DNA [53]. Comparative sequence analysis of 14 neisserial genome sequences from the public domain data [42] showed that *recG*_*Nm*_ harbours five DNA uptake sequences (DUS) in the coding sequence with additional two DUS in the immediate upstream region and one DUS in the immediate downstream region (Fig 9A), making it the DUS-richest Nm gene recognized. Among the 5 DUS present in the coding region, three were located in the wedge domain and two were located in the immediate vicinity of the helicase motifs Ib and IV.

Database searching of the available Nm genomes also identified 49 single nucleotide polymorphisms (SNPs) in *recG*_Nm_, and 37 of them were non-synonymous SNPs (nsSNPs) in the predicted *recG*_Nm_ (Fig 9B). Seven of the nsSNPs are located in the codons for conserved active site residues of RecG_Nm_, including in the wedge, ATP-binding and C-terminal helicase domains (Fig 9B). Using SNAP2 to predict functional effects of the nsSNPs [54], it appears that amino acid substitutions at positions 17, 342, 344 and 438 (S4 Table) may alter RecG function, while the remaining 33 nsSNPs are predicted to be functionally neutral or conservative.

The RecG_Nm_ three-dimensional structure was modelled and SNPs mapped onto the molecular surface (Fig 9C). RecG_Nm_ has typical helicase domains linked to a ´wedge´ domain and shows very little variation in the helicase motifs, with only one parsimony-informative site.

### The Nm wildtype and Δ*recG* mutant strains show unique protein expression profiles

**Fig 10:**
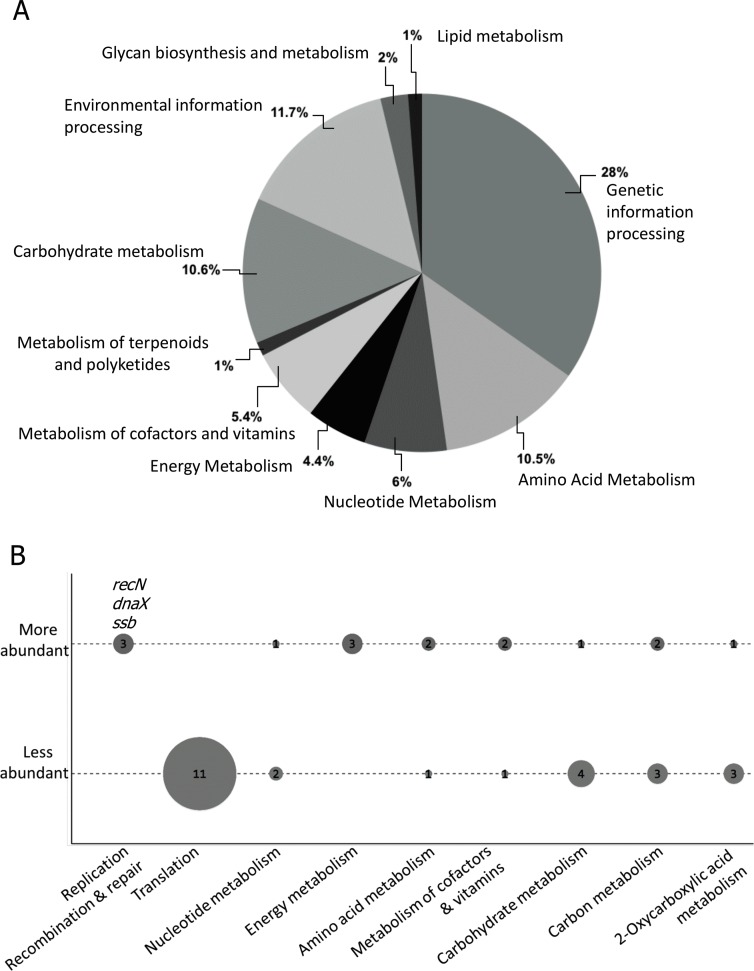
Functional classification of proteins identified and differentially expressed in *Neisseria meningitidis* by mass spectrometry. A. Pie-chart representing the functional classification of all identified proteins identified using high-resolution mass spectrometry. The 1073 proteins were distributed in 10 functional categories based on KEGG orthology using BlastKOALA. B. Differentially more abundant and less abundant proteins in Nm wildtype and NmΔ*recG* sorted by KEGG where the dot plot size is proportional to the counts of differentially expressed (DE) proteins.

In order to identify genes that might be co-regulated with *recG*, the protein expression signatures of Nm wildtype and *ΔrecG* cells were evaluated by mass spectrometry (Fig 10A). In the Nm MC58 wildtype, 1060 proteins were identified while 1064 proteins were identified in Nm MC58 Δ*recG* (Fig 10A). A list of all Nm differentially expressed (DE) proteins is given in S5 and S6 Tables. Relative to wildtype, 83 proteins were DE (29 upregulated and 54 downregulated) in the Δ*recG* strain (Table 2). The type 4 pilus structural subunit protein PilE and the minor pilin protein PilX (NMB0889) were significantly downregulated in Nm *ΔrecG*, while other pilus biogenesis components (PilF, PilT and PilQ) were downregulated to a lesser extent (Table 2). Using BlastKOALA, 43 of the 83 DE proteins could be functionally categorized based on KEGG orthology (Fig 10B). The category of 3R proteins included RecN, SSB, DnaX, and the site-specific recombinase Gcr which were upregulated in the *ΔrecG* mutant (Fig 10B and S6 Table). In addition, superoxide dismutase [Cu-Zn] (SodC) and the universal stress protein (USP, NMB1500) were upregulated in the Nm MC58 *ΔrecG* mutant (S6 Table). The remaining 11 downregulated proteins were mainly ribosomal components involved in translation (Fig 10B).

**Table 2:** The list of differentially expressed proteins involved in DNA replication, recombination and repair and neisserial type IV pilus biogenesis.

Protein fold	Protein name	*Gene name*
	**Replication, recombination and repair proteins**	
2,58	DNA repair protein RecN	recN*
1,36	DNA polymerase III, subunits gamma and tau	dnaX*
3,62	Site-specific recombinase	gcr*
1,75	Single-stranded DNA-binding protein	Ssb*
-3,60	DNA helicase	*uvrD*
-3,35	UvrABC system protein B	*uvrB*
-1,41	Regulatory protein RecX	*recX*
-1,38	DnaA-related protein	NMB1076
-1,24	ATP-dependent DNA helicase RuvA	*ruvA*
-1,20	DNA polymerase III, delta subunit	*holA*
-1,18	DNA polymerase I	*polA*
-1,15	DNA recombination protein RmuC homolog	*rmuC*
-1,12	RecBCD enzyme subunit RecC	*recC*
-1,06	DNA gyrase subunit B	*gyrB*
-1,05	Recombination-associated protein RdgC	*rdgC*
-1,05	DNA polymerase III subunit beta	*dnaN*
-1,01	Protein RecA	*recA*
1,02	UvrABC system protein A	*uvrA*
1,10	DNA polymerase III, epsilon subunit	*dnaQ-2*
1,16	Putative ATP-dependent RNA helicase	NMB1422
1,16	DNA polymerase III subunit alpha	*dnaE*
1,18	Replicative DNA helicase	*dnaB*
1,18	DNA gyrase subunit A	*gyrA*
1,24	ATP-dependent DNA helicase RuvB	*ruvB*
1,30	MutT protein	*mutT*
1,47	DNA topoisomerase 4 subunit A	*parC*
1,51	DNA topoisomerase 1	*topA*
1,68	DNA mismatch repair protein MutS	*mutS*
2,66	RecBCD enzyme subunit RecD	*recD*
4,44	DNA topoisomerase 4 subunit B	*parE*
	**Type 4 pilus biogenesis components**	
-2,69	Fimbrial protein	pilE*
-3,67	Type 4 pilus assembly protein	NMB0889 (pilX)*
-1,38	Twitching motility protein PilT	*pilT-1*
-1,13	Putative type 4 pilus assembly protein PilZ	NMB0770
-1,05	Type 4 pilus biogenesis and competence protein PilQ	*pilQ*
1,01	Twitching motility protein PilT	*pilT-2*
1,09	PilO protein	*pilO*
1,14	PilM protein	*pilM*
1,19	PilN protein	*pilN*
1,31	PilP protein	*pilP*
1,44	Pilus assembly protein PilG	*pilG*
1,88	Type IV pilus assembly protein	*pilF*
-1,03	Twitching motility protein	NMB0051

The minus sign of the protein fold change indicate the downregulated whereas the positive sign shows upregulated proteins. Protein fold changes are log2-transformed t-test difference values.

* Significantly downregulated proteins in Nm in *recG* mutant

### RecG directly interacts with SSB

**Fig 11:**
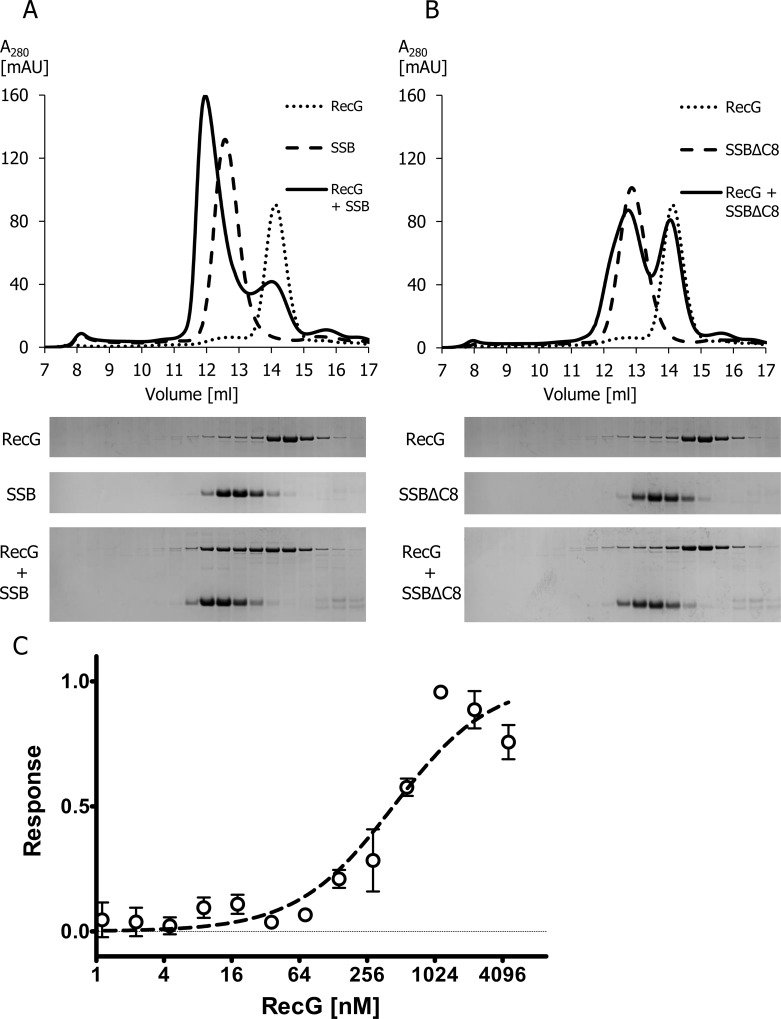
RecG_Nm_ directly interacts with SSB_Nm_. A. Co-gel filtration analysis was performed to monitor the interaction between of RecG_Nm_ and SSB_Nm_. Upper panels: chromatogram A_280_ [mAU] vs retention volume [ml]. Lower panels: SDS-PAGE of 13μl sample from each 0.5ml fraction beginning from 9.5 ml up to 16 ml, and stained with Coomassie blue. A. 20 μM RecG_Nm_ mixed with 40 μM SSB_Nm_ and each protein alone. B. 20 μM RecG_Nm_ mixed with 40 μM SSB _NmΔC8_ and each protein alone. C. Microscale thermophoresis (MST) analysis of the interaction between RecG_Nm_ and SSB_Nm_. MST results of three independent experiments were included. The average and standard deviation of the normalised response and the fitted curve are shown. The calculated Kd value of the interaction is 558 ±139 nM.

Gel filtration chromatography was used to test if there was an interaction between RecG_Nm_ and SSB_Nm_. For this experiment, RecG was pre-incubated with 2X molar excess of SSB prior to gel filtration chromatography on Superdex 200 (Fig 11). Fractions were collected and analyzed by SDS-PAGE (Fig 11, lower panel). When RecG_Nm_ and SSB_Nm_ were mixed before injection on the column, a new peak of the RecG_Nm_:SSB_Nm_ complex appeared that eluted from ≈ 11.2 to 13 ml earlier than the individual peaks alone; and the SSB_Nm_ tetramer [55] eluted between 12 to 13.5 ml buffer (Fig 11A), indicating that RecG_Nm_ and SSB_Nm_ directly interact. Former reports showed that SSB interaction with RecG in *E*. *coli* is mediated by the last eight C-terminal amino acid residues [56,57]. Thus, when performing the same experiment with SSB_Nm_ that lacks the last eight C-terminal amino acids (SSB_NmΔC8_), the peak formed by the RecG_Nm_:SSB_Nm_ complex (Fig 11A) was significantly reduced in the RecG_Nm_:SSB_NmΔC8_ complex (Fig 11B). The reduced RecG_Nm_ and SSB_NmΔC8_ interaction confirmed the necessity of the 8 C-terminal residues of SSB_Nm_ for the interaction with RecG_Nm_. The interaction between RecG_Nm_ and SSB_Nm_ was also confirmed by MST, with SSB_Nm_ as the labelled molecule and RecG_Nm_ as the ligand, indicating a K_d_ value of 558 ±139 nM for this reaction (Fig 11C).

## Discussion

RecG is a double-stranded DNA translocase and helicase thought to play multiple roles in cellular processes including initiation of origin-dependent DNA replication, remodelling, regressing and restarting replication forks stalled at DNA lesions [22,58]. Previous studies show that RecG binds and unwinds a variety of branched model substrates that resemble stalled DNA replication, repair and recombination intermediates [7,20,59]. *In vitro*, *E*.*coli* RecG preferentially unwinds a fork-like DNA substrate with a single-stranded leading arm [22,60], where re-modelling of branched intermediates by RecG through homologous recombination plays a fundamental role in directing DNA synthesis and thus maintaining genomic stability [61]. This study shows that RecG_Nm_ binds and unwinds HJ structure, replication forks and D-loops in the presence of ATP, which is consistent with the proposed roles of RecG_Nm_ in DNA repair, DNA replication and homologous recombination [13,60,62]. Similar to the *E*. *coli* RecG, RecG_Nm_ displayed ATPase activity, however, with equivalent efficiency of ATP hydrolysis with either of the DNA cofactors employed (Fig 4). The preferred co-factor for the *E*. *coli* RecG ATPase activity is negatively-supercoiled DNA for the unbranched DNA [24] and HJ for branched DNA substrates [24,56,63].

We have shown that more than one third of the colonies formed by the Nm *ΔrecG* null mutant are small and non-agglutinating (Fig 5B and 5C), suggesting that this mutant has a lower growth rate than the wildtype. Consistent with this, Sechman et al (2006) showed that both the RecG and RuvABC HJ processing pathways are required for recombinational repair and for normal growth when RecA is expressed [14]. The ribosome efficiency and the amount of ribosomal protein per genome decreased with decreasing growth rate in an *E*. *coli* universal stress protein (USP) mutant [64]. This result in Nm was supported by the mass spectrometry profiling of the *ΔrecG* mutant compared to the wildtype. In fact, in Δ*recG*_Nm_ cells, 11 ribosomal proteins were less abundant as compared to the wildtype (Fig 10B), which might explain the growth defect. Also, the *E*. *coli* USP homologue NMB1500 was significantly upregulated in the Nm *ΔrecG* mutant (S5 Table), and *E*. *coli* USP was shown to be induced in response to stress causing cell growth-arrest [65]. The reduced expression of the type 4 pilus structural subunit protein PilE, PilX as well as the type 4 pilus biogenesis components (PilF, PilT and PilQ) in Δ*recG*_*Nm*_ mutant cells is also consistent with the non-agglutinating colony morphology and phenotypes observed. Nm mutants of the *pilQ* [30], *pilE* [66], *pilT* [67] and *pilG* [68] genes were reported to be transformation deficient. The minor pilin protein PilX is involved in Nm pathogenesis, essential for aggregation and adhesion to host tissues [69].

The initiation of additional replication forks in *E*. *coli* Δ*recG* mutants leads to head to head collision of forks moving in opposite directions, which in turn contributes to the formation of secondary replication forks [10]. This cycle of generating new replication forks results in the accumulation of branched DNA intermediates which interfere with normal DNA replication and make cells defective in chromosome segregation [10]. We hypothesized that in neisserial *ΔrecG* mutant cells, their DNA replication is hindered due to the accumulation of branched DNA structures, which would leave the cells with a reduced number of fully replicated chromosomes compared to the wildtype. However, in this study, flow cytometry assay showed no significant difference with regards to the number of active replication forks between Ng wildtype and *ΔrecG* mutant cells (S7 Fig) and no biologically detectable role for RecG_Nm_ in replication was found.

The Nm *ΔrecG* mutant was sensitive to paraquat, MMS, mitomycin C and UV radiation, but relatively insensitive to hydrogen peroxide, as are *Neisseria* species in general [70]. Although both H_2_O_2_ and paraquat cause oxidative damage, the damage due to paraquat is more severe than the damage caused by H_2_O_2_ [71]. This is because paraquat not only causes oxidative damage via O_2_^-^, but also keeps it on the cycle of production of HO^-^ from H_2_O_2_ by increasing the availability of Fe^2+^ [72]. RecG seems to play a vital role when exposed to paraquat as opposed to hydrogen peroxide. The resistance of Nm *ΔrecG* to H_2_O_2_ might be attributed to the upregulation of Gcr, RecN and SodC (S6 Table) [73]. Unlike the Nm *ΔrecG* mutant, an Ng *ΔrecG* mutant in a previous study was susceptible to H_2_O_2_ exposure [74]. In a former microarray analysis of Ng wildtype exposed to H_2_O_2_, *recN* was the sole gene upregulated compared to other DNA-repair and recombination enzymes [74]. Site-specific recombinases are involved in the control of gene expression, generation of genetic diversity, and separation of dimeric chromosomes; in fact, HJ is the main intermediate for their function [53]. The periplasmic or outer-membrane anchored protein SodC is hypothesized to protect pathogenic bacteria from reactive oxygen species (ROS) of the outside sources, from immune cells [75].

UV-irradiated *E*. *coli* Δ*recG* mutant cells form replication forks outside the origin of replication (oriC) [76], and *recG*-deficient *E*. *coli* cells are sensitive to fork-blocking agents [77,78]. Nm *ΔrecG* cells were sensitive to both MMC and MMS (Fig 8). In *E*. *coli*, alpha-ketoglutarate-dependent dioxygenase (AlkB) promotes repair of alkylation damage to DNA [79]. However, an Nm homolog of AlkB has not been identified [2]. The binding and unwinding of the RecG_Nm_ to model substrates that mimics an arrested DNA replication fork may suggest that RecG_Nm_ also promotes the rescue of alkylation induced replication fork arrest. When a replication fork is arrested, a single strand gap is introduced into the leading strand which is an ideal substrate for RecG [22]. *E*. *coli recG* mutants are sensitive to MMS [80] and the loss of *recG* can be complemented by *recG* from *Mycobacterium tuberculosis*, restoring the ability to repair MMS-, MMC- and UV-induced DNA damage [59].

In our hands, Nm *ΔrecG* cells were extremely sensitive to UV-irradiation with 20-fold lower survival rate than wildtype cells (Fig 8B). This finding conflicts with an earlier report on *E*. *coli* where inactivation of RecG resulted only in moderate sensitivity to UV [78]. This difference might be explained by the fact that *E*. *coli* expresses photolyase, while the *Neisseria* species do not [81]. DNA photolyases are monomeric, light-driven enzymes dedicated to revert lethal UV light-induced DNA damage [82,83]. This would explain the greater role of *recG* in protecting the Nm genome against UV-induced DNA damage. Tønjum and colleagues [2] suggested that nucleotide excision repair (NER) might be the main pathway for repair of UV-induced DNA lesions in *Neisseria*. They also showed that the Nm *uvrA* mutant exhibits a 30,000-fold lower survival than wildtype after exposure to 20 J/m^2^ UV-irradiation [84]. UvrA is part of the endonuclease system involved in the nucleotide excision repair pathway together with UvrB and UvrC [85,86]. It is conceivable that RecG facilitates the repair of DNA lesions by NER by regressing a stalled fork and allowing NER enzymes access to a DNA lesion within duplex DNA [60,87,88]. Hence, it would be interesting to study the ability of NER-deficient RecG mutants to recover from exposure to UV.

As previously reported [14,89], reduced competence for transformation has been observed in MC58*ΔrecG* mutants (Fig 6) when the cells were exposed to DNA for a limited time (15 min). With continuous exposure to DNA over longer time, *ΔrecG* mutant cells demonstrated comparable transformability with the wildtype strain (S6 Fig). Equivalent transformability of *ΔrecG* mutant cells and wildtype cells when the DNA incubation lasted for more than 30 min, might be due to other helicases can compensate the *recG* function as a back-up during genetic transformation; this also suggests that the reduced transformability of Δ*recG*_Nm_ cells at 15 min exposure was not due to a viability problem. Transformation in Ng was severely lowered in the double mutants of *recG* and *ruvA* or *ruvB* strains, whereas *ruvA* and *ruvB* double mutants were transformable equivalent to the wild type [14]. Hence, it is tempting to speculate that RecG_Nm_ is a preferred enzyme within the recombinational repair pathway. Consistent with this idea, it has been reported that the precise structure of a stalled replication fork dictates the kinetics of restart and repair [56]. For instance, when the stalled replication fork includes an exposed region of ssDNA, the SSB first loaded on to ssDNA. The RecG is then recruited prior to recruiting RuvAB [56]. In this context, we have shown that RecG_Nm_ directly interacts with SSB (Fig 11), and SSB is more abundant in the *ΔrecG* mutant than the wildtype as assessed by MS data (S6 Table).

This study also shows that *recG*_Nm_ harbors an unusually high density of DUS [53], rendering *recG* with the highest number of DUS inside a single gene (Fig 9A), while *mutY* is the most DUS-dense gene recognized in terms of number of DUS per nucleotides [52]. It has been observed that the number and density of DUS is significantly higher in neisserial genes involved in DNA repair, recombination, restriction‐modification and replication than in any other gene group [52]. This is consistent with the idea that DUS enhance the probability of DNA uptake, and that there might be selective advantage in efficient uptake of a gene involved in genome maintenance and the response to genotoxic stress [52].

A somewhat unexpected finding was the presence of a high number of nsSNPs in *recG*_Nm_ where one fifth of the nsSNPs were located in functional motifs, including those encoding the wedge, ATP-binding and C-terminal helicase domains. This might contribute to the RecG_Nm_ adaptive potential. In contrast, *M*. *tuberculosis* RecG [90] is highly conserved and very few SNPs in *recG*_*Mtb*_ have been reported to date.

Collectively, these studies on Nm RecG provide insight into its role in DNA repair, recombination and the induction of a phenotypically detectable growth defect, while its potential function in replication requires additional studies.

## Supporting Information

S1 Fig Purification of 6xhis-tagged Nm RecG. Lanes 1, Marker SeeBlue® Plus2 standard, 2, lysate of uninduced bacterial culture, 3, induced control cell lysate (0.5mM IPTG), 4, whole cell lysate, 5, pellet of whole cell lysate, 6, cleared lysate, 7 & 8, 2x wash with 20mM imidazole, 9, wash with 40mM imidazole, 10, flow-through of an Ni^2^-NTA-agarose column, 11–16, elution from Ni^2^-NTA-agarose with 80mM, 120mM, 160mM, 200mM (2x) and 250mM imidazole, respectively.
(TIF)

S2 Fig Quantitation of the gel images of the *Neisseria meningitidis* RecG DNA unwinding assay. A. Holliday junction. B. Fork substrates. The data presented are the means of ± SD from 3 independent experiments.
(TIF)

S3 Fig RecG_Nm_ binds D-loop substrates. Representative gel images from 3 independent experiments. i) 5′ hairpin tail D-loop, ii) 5′ tail D-loops, and iii) 3′ tail D-loop. Lanes: 1) no protein, 2) 200 nM RecG_Nm_, 3) 400 nM RecG_Nm_, 4) 400 nM RecG_Nm_K294A.
(TIF)

S4 Fig RecG_Nm_ unwinds D-loops. Representative gel images from 3 independent experiments. i) 5′ hairpin tail D-loop, ii) 5′ tail D-loop, and iii) 3′ tail D-loop DNA substrates. Lanes: 1) no protein, 2) 200 nM RecG_Nm_, 3) 400 nM RecG_Nm_, 4) 400 nM RecG_Nm_K294A. The Δ designates boiled substrate.
(TIF)

S5 Fig Gel images of ATPase activity of RecG_Nm_ and RecG_Nm_K294A from three independent experiments. A. i) AM13mp18 ssDNA and ii) pET28b(+) dsDNA. B. i) Single-stranded 80 nucleotides polyT. ii) Double-stranded 80 nucleotides polyAT. C. i) Single-stranded 100 nucleotide polyT. ii) Double-stranded 100 nucleotides polyAT. (–) is reaction with no protein, (wt) is wildtype protein (RecG_Nm_), (K294A) is RecG_Nm_K294A.
(TIF)

S6 Fig *Neisseria meningitidis* wildtype and Δ*recG* mutant cells exhibit equivalent DNA transformation frequencies with exposure to DNA for 30 min. Quantitative transformation of *N*. *meningitidis* MC58 wildtype and Δ*recG* mutant with DUS-containing plasmid DNA. The standard deviations of the median from four independent experiments are indicated by bars. Three replicates were inoculated from each sample.
(TIF)

S7 Fig Flow cytometry analysis of *Neisseria gonorrhoeae* cells. Flow cytometry of Hoechst-stained, fixed bacterial cells was performed. For each histogram, the x-axis shows fluorescence levels, which indicates the amount of DNA content per particle counted. The y-axis shows counts, which indicates the number of fluorescing particles or cells. The overlay of sub-population of cells (shaded in black) acquired by gating cells with fluorescence level corresponding to chromosome equivalents of 2, 4, 6, 8, 10 and 12, and the parental histogram (contour). Genome equivalents were determined from the stationary phase and rif-treated *E*. *coli* and are shown in the lower panel (panel V). The X-axis designates the fluorescence intensity in the blue channel, representing the amount of DNA per particle counted. i) *Neisseria gonorrhoeae* (g) MS11 wildtype strain and iii) Ng MS11Δ*recG* mutant strain from the exponential culture, and ii) Ng MS11 wildtype and iv) Ng MS11Δ*recG* mutant strains continued to grow for additional six hours in the presence of 40 μg ml^-1^ rifampicin and 4 μg ml^-1^ cephalexin. V) Slowly growing *Escherichia coli* CM735 stained with Hoechst 33258 was used as standard to calibrate the flow cytometer, as the *E*. *coli* 4.6 Mb chromosome is similar to an Ng diplococcus of 2.3 Mb chromosomes.
(TIF)

S1 Table The list of primers employed in the study. (DOCX)

S2 Table DNA Oligonucleotides employed in this study. (DOCX)

S3 Table *Neisseria meningitidis* does not exhibit a defect in replication. The DNA content and cell mass of individual *Neisseria gonorrhoeae* wildtype and Δ*recG* mutant cells derived from flow cytometry analysis.
(DOCX)

S4 Table *Neisseria meningitidis* amino acid variation. The position of amino acids encoded by non-synonymous single nucleotide polymorphisms (nsSNPs) identified in the deduced RecG protein of *Neisseria meningitidis*.
(DOCX)

S5 Table Significantly down-regulated proteins in *Neisseria meningitidis* MC58 Δ*recG*. Differentially less abundant proteins in *Neisseria meningitidis* (Nm) MC58 Δ*recG* as compared to the Nm MC58 wildtype, sorted according to fold change. Protein fold changes are log2-transformed t-test difference values.
(DOCX)

S6 Table Significantly up-regulated proteins in *Neisseria meningitidis* MC58 Δ*recG*. Differentially more abundant proteins in *Neisseria meningitidis* (Nm) MC58 Δ*recG* as compared to the Nm MC58 wildtype, sorted according to fold change. Protein fold changes are log2-transformed t-test difference values.
(DOCX)
