# Supplementary figures and images for: Characterization of the Neisseria meningitidis Helicase RecG
Source: PLoS One. 2016 Oct 13;11(10):e0164588. doi: 10.1371/journal.pone.0164588 (PMC5063381; doi:10.1371/journal.pone.0164588)

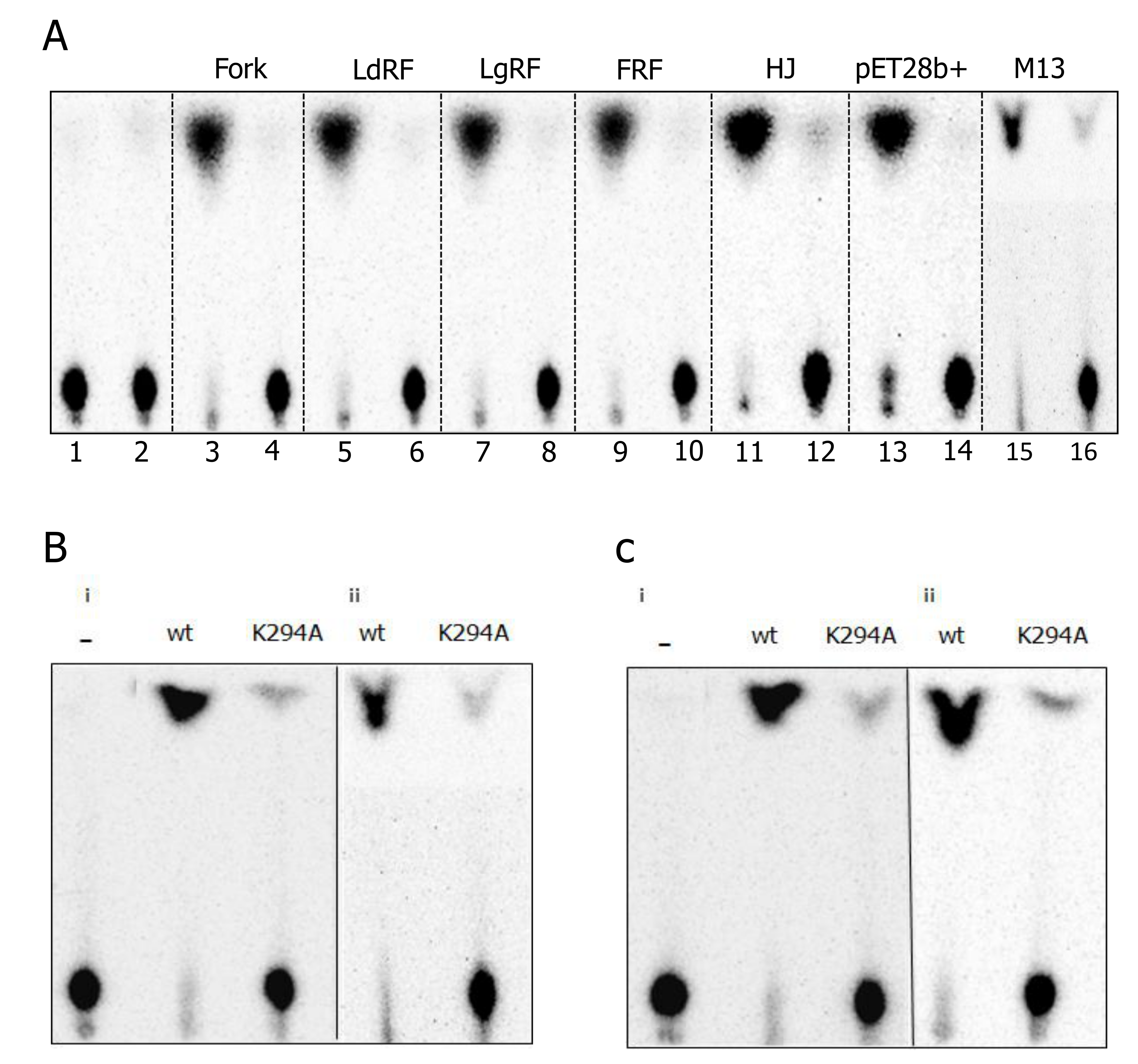

Supplement: S5 Fig — A. i) AM13mp18 ssDNA and ii) pET28b(+) dsDNA. B. i) Single-stranded 80 nucleotides polyT. ii) Double-stranded 80 nucleotides polyAT. C. i) Single-stranded 100 nucleotide polyT. ii) Double-stranded 100 nucleotides polyAT. (–) is reaction with no protein, (wt) is wildtype protein (RecGNm), (K294A) is RecGNmK294A. (TIF) [file pone.0164588.s005.tif]

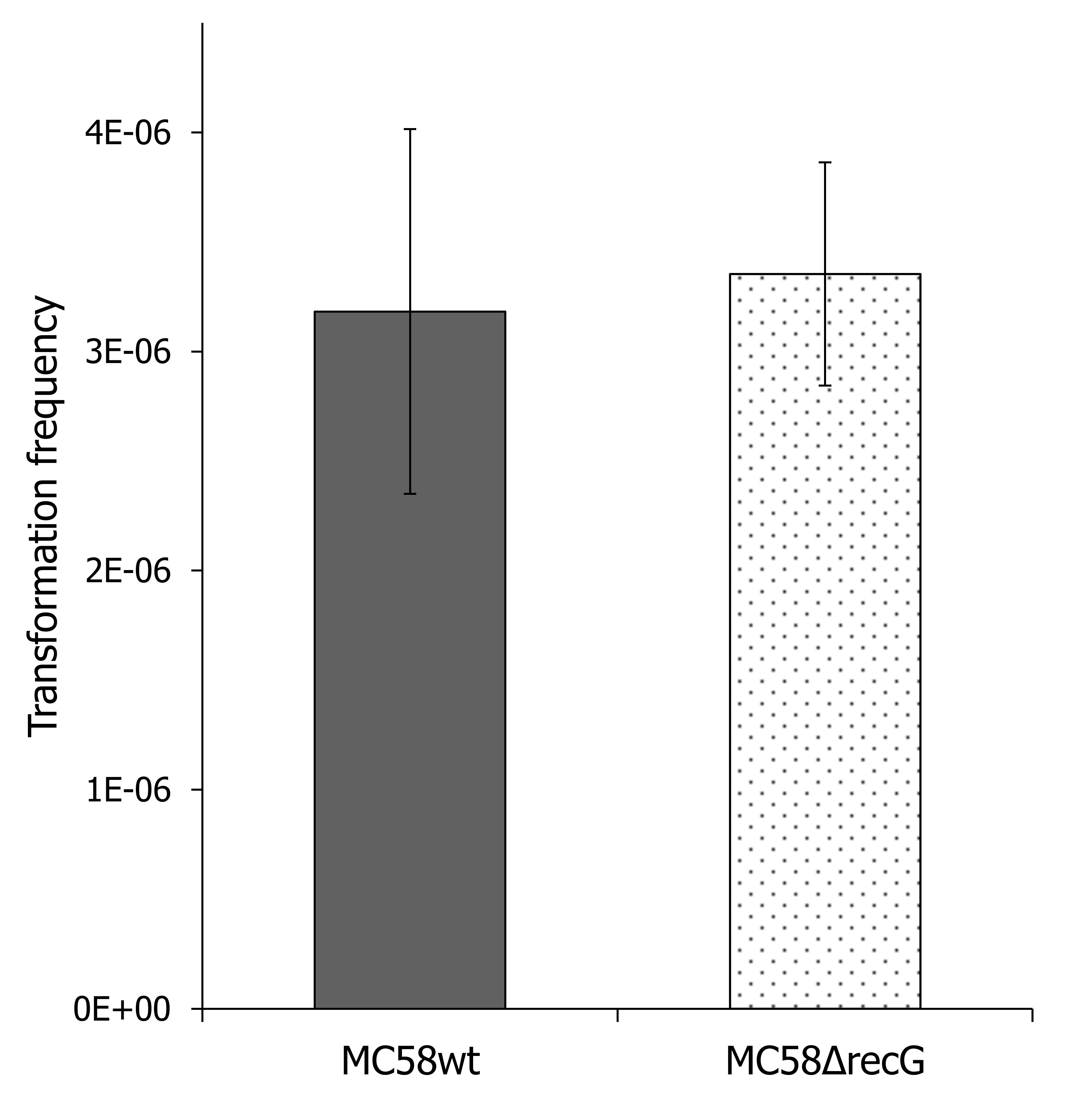

Supplement: S6 Fig — Quantitative transformation of N. meningitidis MC58 wildtype and ΔrecG mutant with DUS-containing plasmid DNA. The standard deviations of the median from four independent experiments are indicated by bars. Three replicates were inoculated from each sample. (TIF) [file pone.0164588.s006.tif]
