# Supplementary material for: Characterization of the Neisseria meningitidis Helicase RecG
Source: PLoS One. 2016 Oct 13;11(10):e0164588. doi: 10.1371/journal.pone.0164588 (PMC5063381; doi:10.1371/journal.pone.0164588)
Supplement: S1 Table — (DOCX) [file pone.0164588.s008.docx]

| Primer  Name | Modification | Lenght (nt) | Sequence *(*5’🡪3’*)* |
| --- | --- | --- | --- |
| *recG* (NMB1788) | | | |
| GTB-3 | NdeI-N-termHis | 35 | CGCATATGATGTCGCCCGAAACCCGAAAACAGCTC |
| GTB-4 | XhoI-for N-termHis | 33 | CGCTCGAGTCACACGCCCAAATACCCTTCCCTG |
| GTB-13 | For sequencing | 20 | CTGTTGCGCCAAAAGTTCAT |
| GTB-14 | For sequencing | 19 | CACGCTTGCGATGAGTTTT |
| GTB-15 | For sequencing | 20 | ACGGCTCAAATTCGATGAAC |
| GTB-16 | For sequencing | 20 | ATCAATGGGCAGACCCAATA |
| RecGK294Afp | Resdigest: BstXI | 43 | GGCGATGTCGGCAGCGGCGCCACCATTGTGGCTGCTTTGTCTG |
| RecGK294Arp | Resdigest: BstXI | 43 | CAGACAAAGCAGCCACAATGGTGGCGCCGCTGCCGACATCGCC |
| SF81 | BamH1 | 28 | GCGGATCCATCAAACACGGCTTTTACGG |
| SF82 | EcoR1 | 28 | GCGAATTCGGCAGATTTCGGAAACAACT |
| SF83 | NheI | 28 | GCGCTAGCCTGCTTTGTCTGCTTTGACG |
| SF84 | HindIII | 28 | GCAAGCTTATCAATGGGCAGACCCAATA |
| *ssb* (NMB1460) | | | |
| KH73 | NheI | 28 | GCGCTAGCGCCGATGAGGATGACTTTGT |
| KH74 | HindIII | 28 | GCGGATCCGCCGTCTATGGTTTCCAAAA |
| KH76 | EcoRI | 28 | GCAAGCTTGCCCCGTAAGGTTTAAGGTT |
| KH75 | BamHI | 28 | GCGAATTCATCCCGAAGTGCGCTATATG |
| SF275 |  | 45 | CCGCCGCCCCGGTCGAGTGAGAGCTCAATTAGCTGAGCTTGGACT |
| SF276 |  | 42 | AAGCTCAGCTAATTGAGCTCTCACTCGACCGGGGCGGCGGCA |

**S1 Table.** The list of primers employed in the study.
