# Supplementary material for: Characterization of the Neisseria meningitidis Helicase RecG
Source: PLoS One. 2016 Oct 13;11(10):e0164588. doi: 10.1371/journal.pone.0164588 (PMC5063381; doi:10.1371/journal.pone.0164588)
Supplement: S2 Table — (DOCX) [file pone.0164588.s009.docx]

| Number | Name | Length | Sequence (5′ 🡪 3′) | Reference |
| --- | --- | --- | --- | --- |
|  |  |  |  |  |
| 1 | HJO1 | 49 | GACGCTGCCGAATTCTGGCTTGCTAGGACATCTTTGCCCACGTTGACCC | [1] |
| 2 | HJO2 | 50 | TGGGTCAACGTGGGCAAAGATGTCCTAGCAATGTAATCGTCTATGACGTT | [1] |
| 3 | HJO3 | 51 | CAACGTCATAGACGATTACATTGCTAGGACATGCTGTCTAGAGACTATCGA | [1] |
| 4 | HJO4 | 50 | ATCGATAGTCTCTAGACAGCATGTCCTAGCAAGCCAGAATTCGGCAGCGT | [1] |
| 5 | RFO1 | 50 | GTCGGATCCTCTAGACAGCTCCATGATCACTGGC ACTGGTAGAATTCGGC | [2] |
| 6 | RFO2 | 50 | CAACGTCATAGACGATTACATTGCTACATGGAGCTGTCTAGAGGATCCGA | [2] |
| 7 | RFO3 | 25 | TAGCAATGTAATCGTCTATGACGTT | [2] |
| 8 | RFO4 | 26 | TGCCGAATTCTACCAGTGCCAGTGAT | [2] |
| 9 | DLO1 | 61 | GGGTGAACCTGCAGGTGGGCGGCTGCTCATCGTAGGTTAGTTGGTAGAATTCGGCAGCGTC | [2] |
| 10 | DLO2 | 61 | GACGCTGCCGAATTCTACCAGTGCCTTGCTAGGACATCTTTGCCCACCTGCAGGTTCACCC | [3] |
| 11 | DLO3 | 41 | TAAGAGCAAGATGTTCTATAAAAGATGTCCTAGCAAGGCAC | [3] |
| 12 | DLO4 | 41 | AAAGATGTCCTAGCAAGGCACGATCGACCGGATATCTATGA | [3] |
| 13 | DLO5 | 61 | TATAGAACATCTTGCTCGTTTTCGAGCAAGATGTTCTATAAAAGATGTCCTAGCAAGGCAC | [4] |

**S2 Table.** DNA Oligonucleotides employed in this study.
