# Supplementary material for: Characterization of the Neisseria meningitidis Helicase RecG
Source: PLoS One. 2016 Oct 13;11(10):e0164588. doi: 10.1371/journal.pone.0164588 (PMC5063381; doi:10.1371/journal.pone.0164588)
Supplement: S3 Table — The DNA content and cell mass of individual Neisseria gonorrhoeae wildtype and ΔrecG mutant cells derived from flow cytometry analysis. (DOCX) [file pone.0164588.s010.docx]

|  | DNA content | SD (DNA content) | total mass | SD (total mass) | DNA/mass | SD  (DNA/mass) | average DNA content | average Mass | average DNA/mass | relative DNA content | relative mass |
| --- | --- | --- | --- | --- | --- | --- | --- | --- | --- | --- | --- |
| MS11 | 276 |  | 154 |  | 1.79 |  |  |  |  |  |  |
|  | 273 | 3.5 | 127 | 13.9 | 2.15 | 0.188 | 276 | 139 | 1.99 | 1.00 | 1.00 |
|  | 280 |  | 135 |  | 2.07 |  |  |  |  |  |  |
| MS11Δ*recG* | 223 |  | 126 |  | 1.77 |  |  |  |  |  |  |
|  | 294 | 3.8 | 159 | 25.9 | 1.85 | 0.22 | 251 | 131 | 1.92 | 0.91 | 0.94 |
|  | 236 |  | 108 |  | 2.19 |  |  |  |  |  |  |
| MS11^++^ | 278 |  | 99.3 |  | 2.80 |  |  |  |  |  |  |
|  | 268 | 9.0 | 90.9 | 4.7 | 2.95 | 0.163 | 277 | 93.9 | 2.95 | 1.00 | 0.68 |
|  | 286 |  | 91.5 |  | 3.13 |  |  |  |  |  |  |
| MS11Δ*recG* ^++^ | 278 |  | 98.4 |  | 2.83 |  |  |  |  |  |  |
|  | 244 | 17.2 | 96.5 | 1.2 | 2.53 | 0.148 | 262 | 97.9 | 2.68 | 0.95 | 0.71 |
|  | 265 |  | 98.8 |  | 2.68 |  |  |  |  |  |  |

**S3 Table. *Neisseria meningitidis* does not exhibit a defect in replication**.The DNA content and cell mass of individual *Neisseria gonorrhoeae* wildtype and Δ*recG* mutant cells derived from flow cytometry analysis.

**^++^** Strains treated with rifampicin and cephalexin
