# Supplementary material for: Characterization of the Neisseria meningitidis Helicase RecG
Source: PLoS One. 2016 Oct 13;11(10):e0164588. doi: 10.1371/journal.pone.0164588 (PMC5063381; doi:10.1371/journal.pone.0164588)
Supplement: S4 Table — The position of amino acids encoded by non-synonymous single nucleotide polymorphisms (nsSNPs) identified in the deduced RecG protein of Neisseria meningitidis. (DOCX) [file pone.0164588.s011.docx]

**

S4 Table .** ***Neisseria meningitidis* amino acid variation.** The position of amino acids encoed by non-synonymous single nucleotide polymorphisms (nsSNPs) identified in the deduced RecG protein of *Neisseria meningitidis*.
