# Supplementary material for: Characterization of the Neisseria meningitidis Helicase RecG
Source: PLoS One. 2016 Oct 13;11(10):e0164588. doi: 10.1371/journal.pone.0164588 (PMC5063381; doi:10.1371/journal.pone.0164588)
Supplement: S5 Table — Differentially less abundant proteins in Neisseria meningitidis (Nm) MC58 ΔrecG as compared to the Nm MC58 wildtype, sorted according to fold change. Protein fold changes are log2-transformed t-test difference values. (DOCX) [file pone.0164588.s012.docx]

**S5 Table. Significantly down-regulated proteins in *Neisseria meningitidis* MC58 Δ*recG.*** Differentially less abundant proteins in *Neisseria meningitidis* (Nm) MC58 Δ*recG* as compared to the MC58 wildtype, sorted according to fold change. Protein fold changes are log2-transformed t-test difference values.

| Protein fold  change | Protein name | Gene name |
| --- | --- | --- |
| -5,81 | Type I restriction enzyme EcoR124II M protein | hsdM |
| -4,74 | Uncharacterized protein | NMB1557 |
| -4,47 | Putative membrane-bound lytic murein transglycylase A | NMB0033 |
| -4,39 | UPF0125 protein NMB0796 | NMB0796 |
| -4,35 | BolA/YrbA family protein | NMB0344 |
| -3,67 | Type IV pilus assembly protein | NMB0889 (pilX) |
| -3,31 | Uncharacterized protein | NMB1024 |
| -2,77 | Uncharacterized protein | NMB1138 |
| -2,70 | Thiol:disulfide interchange protein | dsbA-3 |
| -2,69 | Fimbrial protein | pilE |
| -2,66 | Outer-membrane lipoprotein carrier protein | lolA |
| -2,65 | Putative outer membrane protein OmpH | NMB0181 |
| -2,53 | Polysialic acid capsule biynthesis protein SiaD, truncation  disrupted by foreign ermC cassette | NMB0067 |
| -2,46 | 50S ribomal protein L33 | rpmG |
| -2,46 | Glutathione-regulated potassium-efflux system protein | kefB |
| -2,44 | Lactoferrin-binding protein B | lbpB |
| -2,25 | Peptidylprolyl isomerase | NMB0345 |
| -2,16 | 50S ribomal protein L34 | rpmH |
| -2,11 | Uncharacterized protein | NMB0656 |
| -2,05 | 30S ribomal protein S16 | rpsP |
| -1,84 | GTP cyclohydrolase FolE2 | folE2 |
| -1,76 | Copper-containing nitrite reductase | aniA |
| -1,75 | 50S ribomal protein L27 | rpmA |
| -1,75 | 50S ribomal protein L11 | rplK |
| -1,74 | Phphogluconate dehydratase | edd |
| -1,65 | Major ferric iron-binding protein | fbpA |
| -1,64 | Uncharacterized protein | NMB0036 |
| -1,63 | Putative thioredoxin | NMB1958 |
| -1,63 | 50S ribomal protein L30 | rpmD |
| -1,61 | Glyceraldehyde-3-phphate dehydrogenase | gapA-1 |
| -1,61 | 50S ribomal protein L17 | rplQ |
| -1,60 | Transcription termination factor Rho | rho |
| -1,51 | Dihydroxy-acid dehydratase | ilvD1 |
| -1,51 | Chaperone protein ClpB | clpB |
| -1,51 | Ribonuclease II-related protein | NMB0282 |
| -1,46 | Thiol:disulfide interchange protein DsbC | dsbC |
| -1,44 | Putative lipoprotein NMB1124/NMB1162 | NMB1124 |
| -1,42 | Glutamate-1-semialdehyde 2,1-aminomutase | hemL |
| -1,42 | Acyl CoA thioester hydrolase family protein | NMB0925 |
| -1,41 | Cell division topological specificity factor | minE |
| -1,41 | Glyceraldehyde-3-phphate dehydrogenase | gapA-2 |
| -1,39 | Uncharacterized protein | NMB0283 |
| -1,38 | Cysteine--tRNA ligase | cysS |
| -1,37 | Glutaredoxin | NMB0773 |
| -1,37 | 50S ribomal protein L25 | rplY |
| -1,34 | Orotate phphoribyltransferase | pyrE |
| -1,34 | 50S ribomal protein L6 | rplF |
| -1,30 | Translation initiation factor IF-3 | infC |
| -1,25 | DNA-directed RNA polymerase subunit beta | rpoB |
| -1,25 | 30S ribomal protein S13 | rpsM |
| -1,21 | NifU protein | NMB1380 |
| -1,18 | Aspartate-semialdehyde dehydrogenase | asd |
| -1,16 | L-lactate dehydrogenase | lldD |
| -1,11 | 3,4-dihydroxy-2-butanone 4-phphate synthase | ribB |
