# Supplementary material for: Characterization of the Neisseria meningitidis Helicase RecG
Source: PLoS One. 2016 Oct 13;11(10):e0164588. doi: 10.1371/journal.pone.0164588 (PMC5063381; doi:10.1371/journal.pone.0164588)
Supplement: S6 Table — Differentially more abundant proteins in Neisseria meningitidis (Nm) MC58 ΔrecG as compared to the Nm MC58 wildtype, sorted according to fold change. Protein fold changes are log2-transformed t-test difference values. (DOCX) [file pone.0164588.s013.docx]

| Protein fold  change | Protein name | Gene name |
| --- | --- | --- |
| 4,41 | Putative esterase | NMB1305 |
| 3,94 | Uncharacterized protein | NMB1470 |
| 3,62 | Site-specific recombinase | gcr |
| 3,27 | Uncharacterized protein | NMB1274 |
| 2,97 | Ferredoxin, 4Fe-4S bacterial type | NMB1454 |
| 2,58 | DNA repair protein RecN | recN |
| 2,18 | Lipoprotein | mlp |
| 2,08 | Superoxide dismutase [Cu-Zn] | sodC |
| 2,00 | Trans-sulfuration enzyme family protein | NMB1609 |
| 1,94 | Uncharacterized protein | NMB0086 |
| 1,94 | Aspartate 1-decarboxylase | panD |
| 1,83 | TldD protein | tldD |
| 1,81 | Universal stress protein | NMB1500 |
| 1,75 | Single-stranded DNA-binding protein | ssb |
| 1,68 | Prolipoprotein diacylglyceryl transferase | lgt |
| 1,66 | Agmatinase | speB |
| 1,62 | 2-C-methyl-D-erythritol 2,4-cyclodiphphate synthase | ispF |
| 1,62 | Putative hemolysin activation protein HecB | NMB1762 |
| 1,60 | Riboflavin synthase, alpha subunit | ribE |
| 1,60 | Putative AcnD-accessory protein PrpF | NMB0434 |
| 1,43 | LPS-assembly protein LptD | lptD |
| 1,42 | Uncharacterized protein | NMB1030 |
| 1,41 | Uncharacterized protein | NMB0343 |
| 1,40 | VapD-related protein | NMB1753 |
| 1,40 | Putative amino acid symporter | NMB0194 |
| 1,39 | Ornithine carbamoyltransferase | argF |
| 1,39 | Succinate dehydrogenase iron-sulfur subunit | sdhB |
| 1,36 | DNA polymerase III, subunits gamma and tau | dnaX |
| 1,30 | Phphoribylaminoimidazole-succinocarboxamide synthase | purC |

**S6 Table. Significantly up-regulated proteins in *Neisseria meningitidis* MC58 Δ*recG.*** Differentially more abundant proteins in *Neisseria meningitidis* (Nm) MC58 Δ*recG* as compared to the MC58 wildtype, sorted according to fold change. Protein fold changes are log2-transformed t-test difference values.
